# High-dose Ascorbate Exhibits Anti-proliferative and Anti-invasive Effects Dependent on PTEN/AKT/mTOR Pathway in Endometrial Cancer *in vitro* and* in vivo*

**DOI:** 10.7150/ijbs.102079

**Published:** 2025-01-27

**Authors:** Xiaochang Shen, Jiandong Wang, Weimin Kong, Catherine John, Boer Deng, Shuning Chen, Haomeng Zhang, Jennifer Haag, Nikita Sinha, Wenchuan Sun, Angeles Alvarez Secord, Chunxiao Zhou, Victoria L Bae-Jump

**Affiliations:** 1 Department of Gynecological Oncology, Beijing Obstetrics and Gynecology Hospital, Capital Medical University, Beijing Maternal and Child Health Care Hospital, Beijing 100006, PR China.; 2 Division of Gynecologic Oncology, Department of Obstetrics and Gynecology, University of North Carolina at Chapel Hill, Chapel Hill, NC 27599, USA.; 3 Division of Gynecologic Oncology, Department of Obstetrics and Gynecology, Duke Cancer Institute, Duke University, Durham, NC 27710, USA.; 4 Lineberger Comprehensive Cancer Center, University of North Carolina at Chapel Hill, Chapel Hill, NC 27599, USA.

**Keywords:** endometrial cancer, ascorbate, cell proliferation, invasion, PTEN/AKT pathway, synergy

## Abstract

Endometrial cancer (EC) is the most common gynecological malignancy, frequently characterized by PTEN deletion, activation of the AKT/mTOR pathway, and limited effective treatment options for recurrent and advanced patients. High-dose ascorbate or combined with other chemotherapeutic agents shows potent antitumor effects *in vitro* and *in vivo*. In this study, high-dose ascorbate significantly inhibited cell proliferation and invasion, increased cellular stress and DNA damage, and induced cell cycle arrest and apoptosis in EC cells. Oral or intraperitoneal injections of high-dose ascorbate for 4 weeks effectively inhibited tumor growth in *LKB1^fl/fl^p53^fl/fl^*-mouse model of EC, with intraperitoneal injections being more effective than oral administration. N-acetylcysteine partially reversed the antitumor effects of ascorbate in EC cells and tumor growth in *LKB1^fl/fl^p53^fl/fl^*-mice. PTEN knockdown by shRNA reduced the antitumor sensitivity of EC cells to ascorbate, while inhibition of the AKT/mTOR pathway by Ipatasertib significantly enhanced the antitumor activity of ascorbate in EC cells. Ascorbate combined with paclitaxel synergistically inhibited tumor growth compared to either agent alone in *LKB1^fl/fl^p53^fl/fl^*-mice. Overall, high-dose ascorbate exhibits antitumor activity partially through PTEN/AKT/mTOR and cell stress pathways, and these antitumor effects were heightened when combined with paclitaxel in EC. Clinical trials of ascorbate combined with paclitaxel deserve further investigation in EC patients.

## Introduction

Endometrial cancer (EC) is the most common gynecological cancer, accounting for 58.1% of new diagnoses and 39.1% of deaths from gynecological malignancies in the US in 2024 1. Obesity has been identified as the most relevant risk factor for EC 2. As a result of the rising prevalence of obesity and the aging population, EC has become the only gynecological cancer with an increasing overall incidence, with an estimated global incidence increase of 132% over the past 30 years 2. Endometrioid carcinoma accounts for 85-90% of ECs. The mutation/deletion of the tumor suppressor gene PTEN, leading to the activation of the PI3K/AKT/mTOR pathway, occurs in up to 80% of endometrioid carcinoma 3-5. Most ECs are diagnosed at an early stage and have a good prognosis with total hysterectomy and bilateral salpingo-oophorectomy +/- adjuvant radiation therapy 2, 6. Although patients with recurrent or advanced EC have an approximate 50% response rate to first-line treatment with a carboplatin and paclitaxel doublet, chemoresistance usually develops and subsequently indicates a grim prognosis 2, 7. Hence, innovative therapeutic approaches are highly warranted in EC treatment, especially for advanced and recurrent disease.

Ascorbate, also known as vitamin C, is a well-known essential water-soluble vitamin. Due to the lack of L-gulonic acid-γ-lactone oxidase activity in the body, humans rely on dietary and iatrogenic sources for ascorbate intake 8, 9. Physiological concentrations of ascorbate exhibit antioxidant properties that help to eliminate reactive oxygen species (ROS) and serve as a cofactor of oxygenases, participating in many biological processes such as collagen production, hormone synthesis, and epigenetic and genetic modulation because of its ability to donate electrons 10, 11. Another major function of ascorbate is the protection of vitamin E in the brain. The bodily concentration of ascorbate in the brain is among the highest. There, ascorbate cycles with tocopherol radical regenerating vitamin E therefore supporting its role in preventing brain lipid peroxidation 12. These functions are crucial for maintaining human homeostasis, supporting immune function, and preventing diseases 8, 9, 11, 13. In contrast to physiological concentrations, millimolar levels of ascorbate (also called high-dose ascorbate), only achieved by intraperitoneal (IP) or intravenous administration, have been found to exert potent antitumor effects in pre-clinical models that are dependent on its pro-oxidative activity 8, 14-17. With the existence of endogenous or exogenous metals such as Fe^3+^ or Mn (III) *ortho N*-alkyl- and *N*-alkoxyalkyl porphyrins (MnPs), ascorbate effectively produces large amounts of hydrogen peroxide (H_2_O_2_) intracellularly and subsequently triggers *S*-glutathionylation of cysteines in numerous proteins 8, 14, 16, 18. The results of redox proteomics showed that treating breast 4T1 cancer cells with ascorbate (1 mM) and MnTE-2-PyP^5+^ (5 µM) led to a 1.3-fold or greater increase in oxidation of over 1500 peptidyl cysteines compared with untreated cells, resulting in changes in the regulation of cytoskeleton rearrangement, transcription-mRNA processing, translation, protein folding, cell cycle, and adhesion 18. The combination of ascorbate-induced H_2_O_2_ with other H_2_O_2_ inducers produces significant synergistic effects in inhibiting cell proliferation and tumor growth 19. This has been demonstrated in multiple preclinical models using ascorbate in combination with more than 20 chemotherapeutic agents and radiotherapy 15, 19. Importantly, high-dose ascorbate exhibits no harmful effects on normal cells and tissues and even has the function of protecting normal tissues from damage caused by traditional chemotherapy or radiotherapy. This protection is likely attributed to the presence of low levels of redox-active labile metals and more abundant H_2_O_2_-removing enzymes such as catalase, glutathione peroxidase, and peroxyredoxins 18, 20-23. These promising results have led to clinical trials of intravenous ascorbate as a standalone treatment or in combination with radiotherapy and chemotherapy in cancer patients. Notably, high doses of intravenous ascorbate combined with chemotherapy have demonstrated clinical efficacy and a favorable safety profile in patients diagnosed with ovarian cancer and colorectal cancer 15, 24, 25.

There is limited data on the effects of ascorbate on cell proliferation and tumor growth in EC. Analysis of EC tissues revealed low levels of ascorbate content and this low ascorbate content correlates with a high level of hypoxia-inducible factor-1 (HIF-1) activation and tumor growth, indicating that increasing ascorbate content in EC cells may have an impact on tumorigenesis and development for this disease 26. Therefore, our study aimed to comprehensively investigate the effects of high-dose ascorbate alone and in combination with paclitaxel on inhibiting cell proliferation and invasion in EC cells and a transgenic mouse model of endometrial cancer, including the impact of ascorbate on cellular stress and the PTEN/AKT/ mTOR pathway, as well as its underlying anti-neoplastic potential in EC treatment. Given that obesity is a major risk factor for the carcinogenesis of EC and is associated with a poor prognosis in patients with EC 2, 9, this study also investigated the effects of ascorbate on tumor growth in obese and lean mice induced by a high-fat diet and a low-fat diet, respectively, and compared potential differential responses of obese and lean mice to ascorbate treatment.

## Materials and Methods

### Cell culture and reagents

The human EC cell lines KLE and Hec-1B, Ishikawa, RL-952, AN3CA, and EC-023 were used in this study. KLE, Hec-1B, RL95-2, and AN3CA were purchased from ATCC. Ishikawa cell line was purchased from Sigma. The EC-023 cell line was a gift from Dr. Gertz, University of Utah School of Medicine. The KLE and RL-952 were cultured in DMEM/F12 medium with 10% fetal bovine serum (FBS). The Hec-1B cells were cultured in McCoy's 5A medium with 10% FBS. The Ishikawa were cultured in MEM medium with 5% FBS. The EC-023 were cultured in RPMI 1640 medium with 10% FBS. The AN3CA were cultured in DMEM medium with 10% FBS. All cells were authenticated annually by Labcrop (Burlington, NC). All media was supplemented with 100 units/mL penicillin, and 100 µg/mL streptomycin, and the cells were cultured in an incubator with humidified 5% CO_2_ at 37°C. Of note, both KLE and HEC-1B have wild-type PTEN expression. Sodium ascorbate, MTT, Z-VAD-FMK (Z-V-FMK), N-acetylcysteine (NAC), and paclitaxel (PTX) were purchased from Sigma-Aldrich (St. Louis, MO). Ipatasertib (IPAT) was purchased from MedChemExpress (Monmouth Junction, NJ). The antibodies applied in this article were purchased from Cell Signaling Technology (Beverly, MA) and ABclonal (Woburn, MA), respectively.

### MTT assay

100 µl cell suspensions of KLE, Hec-1B, Ishikawa, RL-952, AN3CA, and EC-023, at concentrations of 4 x 10^4^ - 6 x 10^4^/ml, were added to the 96-well plates and cultured in 5% CO_2_ at 37 °C for 24 hours. The cells were subsequently treated with varying concentrations of ascorbate (from 0.01 to 200 mM) alone or in combination with PTX or IPAT for 72 hours. MTT solution (5 mg/ml) was added to the 96-well plates at 5 µl/well, and the plates were incubated at 37 °C for 1 hour. The supernatants were removed, and 100 µl of dimethyl sulfoxide (DMSO) was added to each well to dissolve the formazan crystal. The results were read by a Tecan microplate reader (Morrisville, NC) at a wavelength of 562 nm. The effects of these agents on cell proliferation were calculated as the percentage of control cells grown in the same 96-well plates. The IC50 of ascorbate in each cell line was calculated by the AAT IC50 calculator (AAT Bioquest; Sunnyvale, CA). The Bliss independence model was used to calculate the synergistic effect as combination index (CI ) < 1 (synergistic), CI = 1 (additive), or CI > 1 (antagonistic) 27.

### Colony formation assay

The KLE and Hec-1B cells were seeded in the 6-well plates at 300 cells/well for 24 hours and then replaced with their regular medium containing ascorbate (0.1, 1, and 5 mM) for 24 hours. After that, the cell lines were continually cultured with medium changes every three days. After 2 weeks of culture, 1 ml 100% methanol was added to the plate to fix the cells for 20 minutes, and then 1 ml 0.5% crystal violet was added to stain the cells for another 20 minutes. The total number of colonies was counted under a microscope.

### Analysis of cell cycle by Cellometer imaging

The KLE and Hec-1B cells were plated at 2.5 x 10^5^ cells/ml in 6-well plates for 24 hours and exposed to 0.1, 1, and 5 mM ascorbate for 36 hours. Cells were harvested with a mix of 0.05% Trypsin-EDTA and 0.25% Trypsin (1:3) (Sigma-Aldrich, St. Louis, MO) and fixed in 100% methanol for 30 minutes. The cells were resuspended in a solution of 10 mM propidium iodide, 50 µg RNase A, and 0.05% Triton X-100 and then incubated at 37 °C in the dark for 30 minutes. Cellometer (Nexcelom, Lawrence, MA) was used to measure cell cycle progression. This system demonstrated comparable performance to flow cytometry in analyzing cell cycle profiles. However, a limitation of this system is that a small fraction of cells (e.g., the Sub-G1 fraction) may not provide sufficient fluorescence intensity for that specific subpopulation, thus affecting the reliability of the results 28. The FCS7 express software (Molecular Devices, Sunnyvale, CA) was used to analyze the data.

### Cleaved caspase 3 ELISA assay

The KLE and Hec-1B cells were plated at a density of 2.5 x 10^5^ cells/ml in 6-well plates for 24 hours. Cells were exposed to 0.1, 1, and 5 mM ascorbate for 6 hours. Following exposure, the cells were lysed using 1X caspase lysis buffer and centrifuged at 4°C and 15,000 rpm for 15 minutes. The supernatant was collected and mixed with a reaction buffer of caspase 3 (200 µM) (AAT Bioquest) at 37°C in the dark for 30 minutes. A Tecan plate reader was used to read the plates with an excitation/emission wavelength of 341/441 nm.

### Adhesion assay

The KLE and Hec-1B cells were seeded in plates pre-coated with laminin-1 (Sigma-Aldrich) at 5,000 cells/well and treated with 0.1, 1, and 5 mM ascorbate for 1.5 hours. The supernatants were aspirated, and the cell lines were fixed with 5% glutaraldehyde for 30 minutes. After that, the plate was stained with 0.1% crystal violet solution for 15 minutes at room temperature, and 100 μl of 10% acetic acid was added to each well to solubilize the dye. The plate was measured at a wavelength of 575 nm by a Tecan plate reader.

### Transwell assay

Cell invasion assays were conducted in 96-well plates pre-coated with 0.5-1 x BME (Trevigen, Gaithersburg, MD). The KLE and Hec-1B cells were starved in an FBS-free medium for 12-24 hours and then plated in the upper chamber with a density of 3x10^4^. 0.1, 1, and 5 mM ascorbate were added to the upper chambers. The lower chambers were filled with regular medium. The plates were then incubated for 2 - 4 hours for cell invasion into the lower chambers. Both chambers were washed with PBS after the incubation. 100 μl of calcein AM solution (Invitrogen, Carlsbad, CA) was added to the lower chambers and incubated for another 30 minutes. The plate was measured using a Tecan plate reader with an excitation/emission wavelength of 485/520 nm.

### Wound healing assay

The KLE and Hec-1B cells were plated at 2.5 x 10^5^ cells/ml in 6-well plates. When the cells merged to over 70-80% confluence, the plate was scratched with two cross lines by a 200 µl sterile pipette tip. The plates were gently washed twice with PBS and then exposed to 0.1, 1, and 5 mM ascorbate for 24 hours. Images were taken by a microscope after 24 hours of treatment. Wound width was analyzed by the ImageJ software (National Institutes of Health; Bethesda, MD).

### ROS assay

Intracellular ROS levels induced by ascorbate were measured by 2,7-dichlorodihydrofluorescein diacetate (DCFH-DA) assay. The KLE and Hec-1B cells (10,000/well) were seeded in 96-well plates for 24 hours and then treated with 0.1, 1, and 5 mM ascorbate for 6 hours. The supernatants were aspirated, and the cells were incubated at 37°C with phenol-red-free regular media containing DCFH-DA (15 μM) for 30 minutes. A Tecan plate reader was used to read the plate with an excitation/emission wavelength of 485/526 nm.

### JC-1 assay

The KLE and Hec-1B cells (15,000/well) were plated in 96-well plates. After 24 hours, the cells were treated with ascorbate (0.1, 1, and 5 mM) for 6 hours. 1 μl of JC-1 (2 μM) was added to each well and incubated for 30 minutes at 37°C in the dark. After washing with PBS, a Tecan plate reader was used to read the plate with an excitation/emission wavelength of 535/590 nm for the red JC-1 signal and 485/535 nm for the green signal.

### TMRE assay

The KLE and Hec-1B cells (2x10^5^/well) were seeded in black 96-well plates for 24 hours. The cell lines were exposed to 0.1, 1, and 5 mM ascorbate for 6 hours. 100 µl of 1 mM TMRE (tetramethylrhodamine ethyl ester) was added to each well for 30 minutes at 37°C in the dark. After washing with PBS, a Tecan plate reader was used to read the plate with an excitation/emission wavelength of 549/575 nm.

### Trolox equivalent antioxidant capacity (TEAC) assay

The antioxidant capacity of cells and serum from mice was measured by TEAC assay. The KLE and Hec-1B cells (2.5 x 10^5^ cells/ml) were seeded in 6-well plates for 24 hours and then exposed to 0.1, 1, and 5 mM ascorbate for 6 hours. The cells were harvested using PBS and lysed using sonication. The cell lysate was centrifuged at 4°C and 12,000 rpm for 15 minutes. After adjustment of concentration based on the bicinchoninic acid (BCA) assay (Bio-Rad, Hercules, CA), the supernatants were added to a 96-well plate with an equal amount and volume of protein (30 µl). Similarly, the serum obtained from mice was diluted at a ratio of 1:100 and added to a 96-well plate with an equal volume (30 µl). 120 µl of ABTS substrate working solution (Sigma-Aldrich) was quickly added to each well, and the plate was incubated on an orbital shaker at room temperature for 5 minutes. A Tecan plate reader was used to detect the plate at a wavelength of 410 nm. The higher OD value represents a lower antioxidant capacity.

### Western immunoblotting

The KLE and Hec-1B cells were seeded in 6-well plates at 2.5 x 10^5^ cells/ml and treated with 0.1, 1, and 5 mM ascorbate. The RIPA buffer was added to lyse the cells for 30 minutes on ice, and the cell lysate was collected and centrifuged at 4°C and 15,000 rpm for 15 minutes. The protein concentration of the supernatants was quantified by a BCA assay. An amount of 20-30 µg protein was loaded per lane and separated by sodium dodecyl sulfate-polyacrylamide gel electrophoresis (SDS-PAGE). The proteins were transferred onto PVDF membranes at 4°C for 90-120 minutes and then the membrane was blocked with 5% fat-free milk for 1 hour at room temperature. The membrane was incubated with a primary antibody at 4°C overnight and incubated with the secondary antibody for 1 hour at room temperature after washing with TBS-T buffer 3 times. The membranes were developed by the Western Lightning Plus-ECL (PerkinElmer, Waltham, MA) on the ChemiDoc Image System (Bio-Rad; Hercules, CA). The quantitative assessment of protein expression was analyzed by Image Lab software and normalized to appropriate internal controls in the same PVDF membrane (Supplementary Figures 2 and 3).

### shRNA transduction

PTEN shRNA lentiviral transduction plasmid (TRCN0000002747) and non-targeting shRNA lentiviral transduction plasmid (pLKO.1-puro Non-Target Control [SHC016V]) were purchased from Sigma-Aldrich. 293 T cells were used for plasmid packaging based on the manufacturer's protocol. After 72 hours of packaging, the supernatants were collected and passed through 0.45 μm filters to remove the cell debris. The Hec-1B cells with wild-type PTEN were used for transfection. The cells were seeded in 6-well plates for 24 hours. When the cells merged to over 40-50% confluence, the virus with 10 μg/mL polybrene was added to each well and incubated for 72 hours. After that, 2 μg/ml puromycin was used to select transfected cells. The efficiency of PTEN transfection was confirmed using Western immunoblotting analysis.

### *Lkb1^fl/fl^p53^fl/fl^*-transgenic mouse model of EC

The *LKB1^fl/fl^p53^fl/fl^*-genetically engineered mouse model of endometrioid EC was created in our lab 29. Recombinant adenovirus Ad5-CMV-Cre (Ad-Cre) (Transfer Vector Core, University of Iowa) at a titer of 10^11^-10^12^ infectious particles/ml was used for intrauterine injection and tumor induction. Given that EC is a highly obesity-driven cancer, we assessed the effects of ascorbate on tumor growth in both obese and lean mice. Four-week-old mice were randomly divided into two groups, one group was given a high-fat diet (HFD, obese group; 60% of calories from fat; Research Diets, New Brunswick, NJ) to induce obesity, and the other group was given a low-fat diet (LFD, lean group; 10% kcal from fat; Research Diets, New Brunswick, NJ). The Ad-Cre virus was injected into the left uterine horn of the *LKB1^fl/fl^p53^fl/fl^*-mice at eight weeks of age to induce EC. Then, the mice were randomly assigned to different groups, with each group containing at least 15 mice. Treatment started ten weeks after the Ad-Cre virus injection, and the mice were weighed weekly throughout the study.

To detect the effect of different administration routes of ascorbate on tumor growth, the HFD and LFD mice were divided into different groups. In the ascorbate oral group, to ensure that each mouse in the oral ascorbate group received the same concentration of ascorbate treatment in both obese and lean conditions, we measured the daily water intake of the obese and lean mice and found that before ascorbate treatment, obese mice consumed an average of 5.9 ml of water per day, while lean mice consumed an average of 4.4 ml of water per day. Besides, the average body weight of mice in the HFD group is 35 g, while the average body weight of mice in the LFD group is 26 g. The concentration of ascorbate in drinking water during subsequent treatments was calculated based on the amount of water consumed and the body weight of the obese and lean mice. Hence, mice were divided into 8 subgroups: HFD-vehicle (Oral), HFD-Ascorbate oral (6.6 g /L, equivalent to 1.11 g/kg/day, with water changed every 2 days), HFD-PTX (6.25 mg/kg, IP, once a week), HFD-Ascorbate + PTX (Ascorbate 6.6 g/L, with water changed every 2 days; PTX 6.25 mg/kg, IP, once a week); LFD-vehicle (Oral), LFD-Ascorbate oral (6.55 g/L, equivalent to 1.11 g/kg/day, with water changed every 2 days), LFD-PTX (6.25 mg/kg, IP, once a week), LFD-Ascorbate + PTX (Ascorbate 6.55 g/L, with water changed every 2 days; PTX 6.25 mg/kg, IP, once a week). In the ascorbate IP injection group, mice were further divided into 4 subgroups: HFD-vehicle (IP), HFD-Ascorbate IP (4 g/kg, twice a day), LFD-vehicle (IP), LFD-Ascorbate IP (4 g/kg, twice a day). To investigate the effect of oxidant stress on the antitumor activity of ascorbate, the *LKB1^fl/fl^p53^fl/fl^*-mice on the LFD were divided into LFD-vehicle (IP), LFD-Ascorbate IP (4 g/kg, twice a day), LFD-NAC (150 mg/kg, once a day, IP), LFD-NAC + Ascorbate (NAC: 150 mg/kg, once a day, IP; Ascorbate 4 g/kg, twice a day, IP). All mice were euthanized after four weeks of treatment. Endometrial tumors and the tissues of the heart, lung, liver, spleen, and kidney were collected and weighted, with one-half of the tissues fixed in 10% neutral-buffered formalin and paraffin-embedded, and the remaining half snap-frozen and stored at -80°C. Blood samples were taken, and serum was stored at -80°C. Animal experiments were approved by the Institutional Animal Care and Use Committee (IACUC, 21-229) of the University of North Carolina at Chapel Hill (UNC-CH).

### Immunohistochemistry (IHC) of endometrial tumor specimens

The mouse endometrial tumor tissues were first fixed with formalin, then embedded with paraffin, and cut into sections (4 μm) at the Animal Histopathology Core Facility at UNC-CH. The slides were deparaffinized with xylene and hydrated with ethanol. An appropriate antigen retrieval buffer was used to boil the slides for 3 min in a pressure cooker, followed by soaking in cold water for 10 minutes. The protein block solution (Dako, Agilent Technologies, Santa Clara, CA) was used to block the slides for 30 minutes at room temperature. The slides were incubated with primary antibodies of Ki-67 (1:400), phosphorylated (p)-S6 (1:800), Bcl-xL (1: 1200), VEGFR-2 (1:2000), BiP (1:800), and PTEN (1:200) overnight at 4°C. The secondary antibodies (biotinylated goat anti-rabbit, Vector Labs, Burlingame, CA) were applied to incubate the slides at room temperature for one hour after washing with washing buffer for 2 minutes x 3 times. ABC Substrate System (Vector Labs) was used for the color reaction, 3,3′-Diaminobenzidine (DAB) was used as a chromogen, and Mayer's hematoxylin was used for counterstaining. All the slides were scanned by Motic (Houston, TX) and scored by ImagePro software (Vista, CA). The H-score (Histochemical score) system was used in this study to assess protein expression. Staining intensity was categorized as 0 (no staining), 1 (weak), 2 (moderate), or 3 (strong), and the percentage of positive cells at each intensity level was determined as P1, P2, and P3, respectively. The formula is: H-score= (1*P1) + (2*P2) + (3*P3). The total score ranged from 0 to 300, with higher scores indicating stronger and more extensive protein expression.

### Hematoxylin and Eosin (H&E) staining of specimens

The heart, lung, liver, spleen, and kidney tissues of mice were first fixed with formalin and then embedded with paraffin. H&E staining was performed at the Animal Histopathology Core Facility at UNC-CH. All the slides were scanned by Motic (Houston, TX) and scored by ImagePro software (Vista, CA).

### Statistical analysis

All data were presented as a mean ± SD. Experiments were repeated three times except for animal experiments. The GraphPad Prism 8 software (La Jolla, CA) was used to calculate all the statistical results and create the graphs. An unpaired Student's t-test was used for comparisons between groups, and one-way ANOVA with Tukey's multiple comparison test was used for comparisons between multiple groups. Two-way ANOVA was used to analyze the differences between non-transfected and transfected EC cells, and HFD and LFD mice. p<0.05 were considered statistically significant.

## Results

### Ascorbate inhibited cell proliferation, tumor growth, and activation of the PI3K/AKT and MAPK pathway

**Figure 1:**
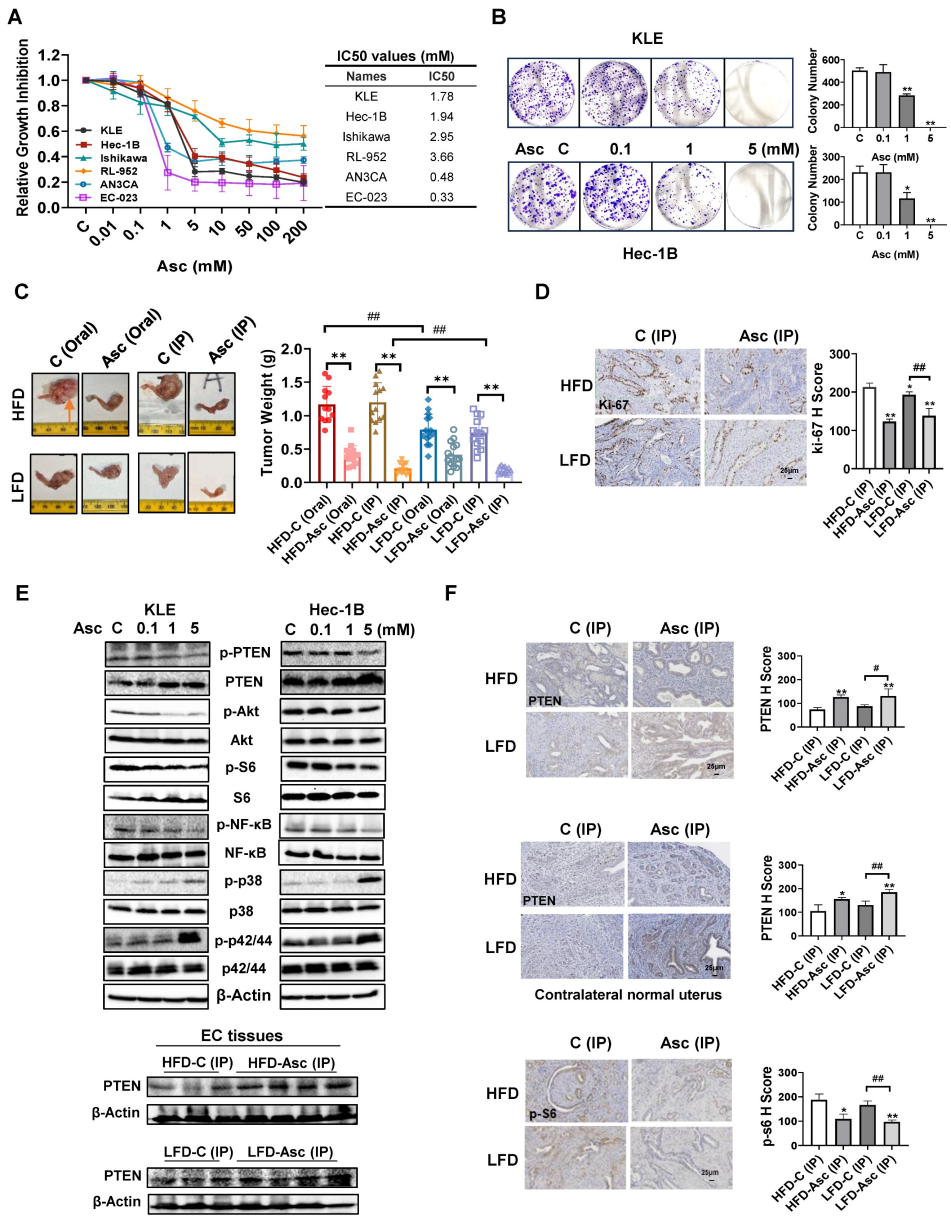
** Ascorbate inhibited cell proliferation and tumor growth in EC cells and the** ***LKB1^fl/fl^p53^fl/fl^*****-transgenic mouse model.** The human EC cell lines KLE, Hec-1B, Ishikawa, RL-952, AN3CA, and EC-023 were treated with ascorbate (from 0.01 to 200 mM) for 72 hours. Cell proliferation was detected by the MTT assay. Ascorbate inhibited the proliferation of all these cells in a dose-dependent manner with varying IC50s (A). Ascorbate inhibited colony formation in KLE and Hec-1B cells compared to control cells (B). The obese and lean *LKB1^fl/fl^p53^fl/fl^*-mice were treated with ascorbate (Oral), ascorbate (IP), or their own vehicle for four weeks, and the results showed that ascorbate effectively reduced tumor weight compared to their own control in both HFD and LFD mice. The symbol (↑) indicates tumor (C). IHC results showed that ascorbate (IP) reduced the expression of Ki-67 in endometrial tumor tissues of *LKB1^fl/fl^p53^fl/fl^*-mice in both HFD and LFD groups (D). Western blotting results showed that ascorbate increased the expression of PTEN, phosphorylated p38, and phosphorylated p42/44 and inhibited the expression of phosphorylated PTEN, phosphorylated AKT, phosphorylated S6, and phosphorylated NF-κB in both cells after treatment for 24 hours. Ascorbate (IP) increased the expression of PTEN in EC tumors in both HFD and LFD groups after treatment for 4 weeks (E). IHC results showed that ascorbate treatment elevated the expression of PTEN and reduced the expression of p-S6 in endometrial tumor tissues, and also elevated the expression of PTEN in the contralateral normal uterus of *LKB1^fl/fl^p53^fl/fl^*-mice in both HFD and LFD groups (F). *p<0.05, **p<0.01 compared with Control; *^#^p*<0.05,*^ ##^p*<0.01 compared with each group; Asc = Ascorbate, C (Oral) = Vehicle control for oral administration group, C (IP) = Vehicle control for IP injection group.

The KLE, Hec-1B, Ishikawa, RL-952, AN3CA, and EC-023 cells were exposed to a range of concentrations of ascorbate (0.01-200 mM) for 72 hours, and cell proliferation inhibition was detected by the MTT assay. The results demonstrated that ascorbate dose-dependently decreased the cell viability of all EC cell lines, with different IC50s for each cell line and statistical differences between some cell lines (Figure 1A, supplementary table 1). Among all 6 cell lines, EC-023 was the most sensitive cell line with an IC50 of 0.33 mM, while RL-952 cell line was the most resistant cell line with an IC50 of 3.66 mM. The KLE and Hec-1B cells were used to further detect the long-term effect of ascorbate on cell proliferation by colony assay. Both cell lines were exposed to 0.1, 1, and 5 mM ascorbate for 24 hours, and then the cells were cultured for another 14 days. Ascorbate at a dose of 1 mM significantly inhibited colony formation by 42.4% in KLE cells and 49.8% in Hec-1B cells, respectively (Figure 1B, p<0.05).

To further investigate different administration routes of ascorbate on tumor growth, the obese and lean *LKB1^fl/fl^p53^fl/fl^-*transgenic mice were treated with ascorbate at 1.11 g/kg by oral administration through drinking water or 4 g/kg twice a day by IP injection for four weeks. Consistent with our previous study 29, 30, HFD significantly increased tumor weight compared with mice fed an LFD (HFD vs LFD: 1.17 g vs 0.75 g in the oral group and 1.20 g vs 0.73 g in the IP group, p<0.01, Figure 1C). Oral administration of ascorbate inhibited tumor growth by 64.6%, and IP injection of ascorbate inhibited tumor growth by 83.3% compared with their own control in obese mice. In the lean mice, oral administration of ascorbate inhibited tumor growth by 44.1% and IP injection inhibited tumor growth by 74.6% compared with their own control mice, respectively (Figure 1C, p<0.05). Obese mice appeared to be more sensitive to ascorbate treatment than lean mice (p<0.05). Treatment of mice with high-dose ascorbate for 4 weeks did not affect body weight compared with control mice (Supplementary Figure 1A and B). These results suggest that IP injection of high-dose ascorbate is more effective than oral administration of ascorbate in inhibiting tumor growth in both obese and lean mice. IHC staining results showed that ascorbate (IP) significantly reduced the expression of Ki-67 by 42.15% and 28.01% in EC tumors in obese and lean groups compared to control mice, respectively (Figure 1D, p<0.01).

Since the PTEN/AKT/mTOR pathway and MAPK pathways are vital for the carcinogenesis and development of EC, the effect of ascorbate on these pathways was investigated by Western blotting in the KLE and Hec-1B (wild-type PTEN) cell lines. After being treated with ascorbate (0.1, 1, and 5 mM) for 24 hours, Western blotting showed that ascorbate increased the expression of PTEN, phosphorylated p42/44, and phosphorylated p38, and significantly decreased the expression of phosphorylated PTEN, phosphorylated AKT, phosphorylated S6, and phosphorylated NF-κB in the KLE and Hec-1B cells (Figure 1E). Furthermore, the Western blotting of the EC tissues obtained from the *LKB1^fl/fl^p53^fl/fl^*-transgenic mouse model also showed that ascorbate (IP) administration increased the expression of PTEN in both obese and lean mice (Figure 1E). IHC staining results showed that ascorbate (IP) significantly increased the expression of PTEN by 70.50% and 49.19% in EC tumors in obese and lean groups respectively, compared to control mice (Figure 1F, p<0.05). Similar results were also observed in the contralateral normal endometrium, as ascorbate (IP) significantly increased the expression of PTEN by 48.21% and 41.35% in obese and lean groups compared to control mice, respectively (Figure 1F, p<0.05). The positive staining of phosphorylated S6 was also decreased by 51.73% and 44.91% in EC tumors compared to control mice in both obese and lean groups, respectively (Figure 1F, p<0.05). These results demonstrated that the function of ascorbate in the inhibition of cell proliferation and tumor growth may be associated with the activity of the PTEN/AKT/mTOR and MAPK pathways in EC.

### Ascorbate induced cell cycle arrest, autophagy, and apoptosis in EC

**Figure 2:**
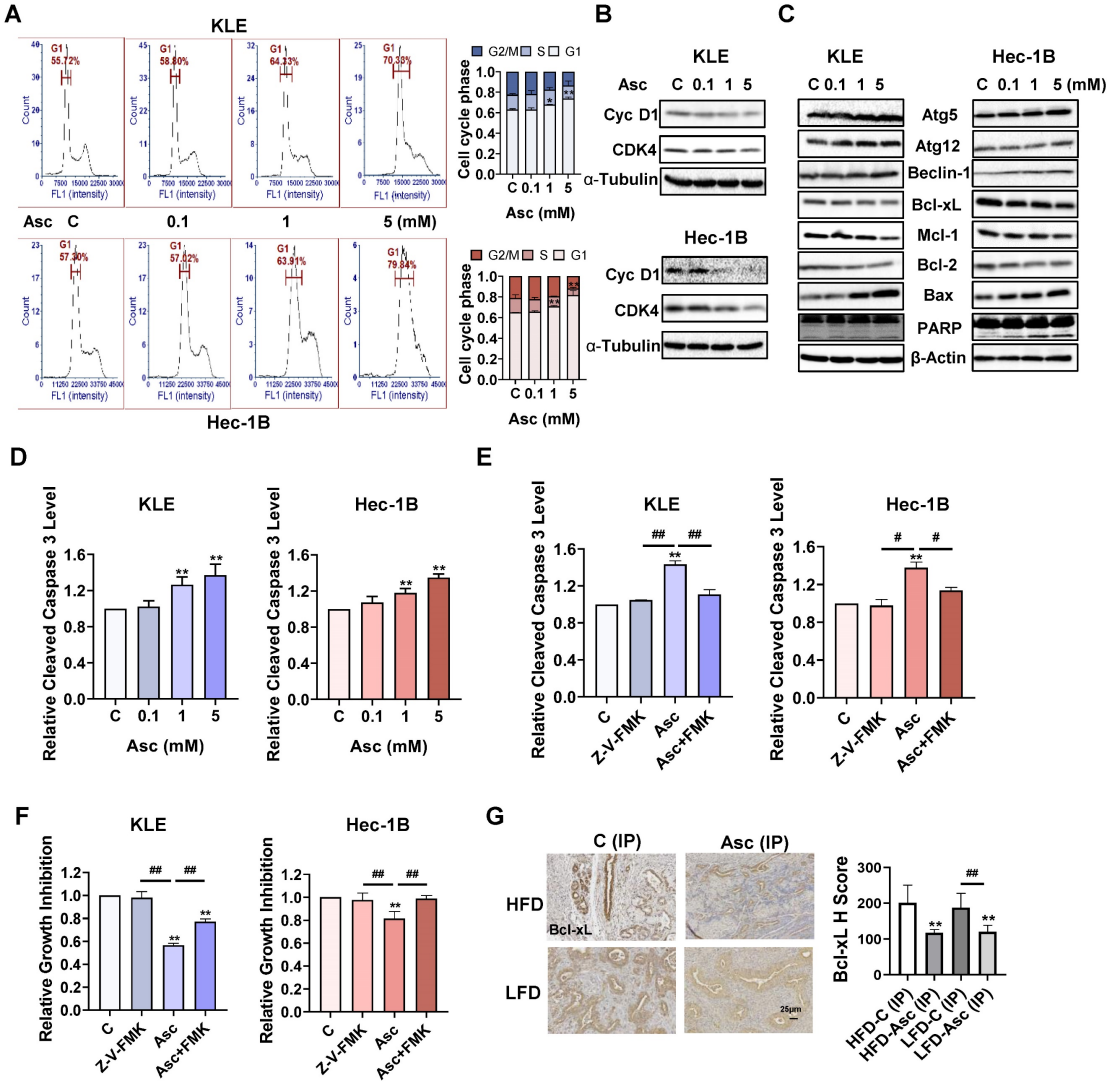
** Ascorbate induced cell cycle arrest, autophagy, and apoptosis in EC.** The KLE and Hec-1B cells were treated with 0.1, 1, and 5 mM ascorbate for 36 hours. The cell cycle profile was assessed by Cellometer. Ascorbate treatment resulted in cell cycle G1 phase arrest in the KLE and Hec-1B cells (A). Western blotting results showed that ascorbate inhibited the expression of CDK4 and cyclin D1 in both cell lines after 24 hours of treatment (B). Western blotting results showed that ascorbate inhibited the expression of Bcl-xL, Mcl-1, and Bcl-2 and increased the expression of BAX, cleaved PARP, Atg5, Atg 12, and Beclin-1 after 6 hours of treatment (C). ELISA assay showed that ascorbate treatment significantly increased the activities of cleaved caspase 3 after 6 hours of treatment (D). Z-V-FMK (10 µM) was used to pre-treat both cells for 2 hours, followed by treatment with ascorbate for 6 hours. Z-V-FMK pre-treatment blocked the increased cleaved caspase 3 level induced by ascorbate (1 mM) (E). The MTT assay showed that Z-V-FMK significantly recovered cell viability induced by ascorbate (1 mM) in both cell lines after 72 hours of treatment (F). IHC results demonstrated that ascorbate reduced the expression of Bcl-xL in endometrial tumor tissues of *LKB1^fl/fl^p53^fl/fl^*-mice in both the HFD and LFD groups (G). *p<0.05, **p<0.01 compared with C. *^#^p*<0.05, *^##^p*<0.01 compared with each group. Asc = Ascorbate, C (IP) = Vehicle control for IP injection group.

To investigate the mechanism by which ascorbate inhibits cell growth, cell cycle profiles were assessed by Cellometer in the KLE and Hec-1B cells after 36 hours of ascorbate treatment. Ascorbate induced the G1 phase arrest in a dose-dependent manner in both cell lines. Ascorbate at a dose of 5 mM increased the G1 phase from 62.95% in the untreated group to 73.83% in the KLE cells and 65.05% to 81.73% in the Hec-1B cells (Figure 2A, p<0.01). Western blotting results confirmed that ascorbate decreased the expression of cyclin D1 and CDK4 in both cell lines after 24 hours of treatment (Figure 2B). Furthermore, Western blotting also demonstrated that the expression of the autophagy-related proteins Atg5, Atg12, and Beclin-1 were upregulated in both cells after 6 hours treatment of ascorbate, indicating that high-dose ascorbate-induced autophagy may be involved in inhibiting cell proliferation of EC cells (Figure 2C).

Because the dysregulation of the cell cycle and cell autophagy may trigger cell apoptosis, the expression of proteins related to apoptosis was analyzed by Western blotting assay. Treatment of both cell lines with 0.1, 1, and 5 mM ascorbate for 6 hours significantly decreased the expression of Bcl-xL, Mcl-1, and Bcl-2, and increased the expression of Bax and cleaved-PARP (Figure 2C). ELISA assay showed that ascorbate (1 and 5 mM) significantly increased the levels of cleaved caspase 3 activity in the KLE and Hec-1B cells after 6 hours of treatment. 5 mM ascorbate increased the levels of cleaved caspase 3 by 37.1% in the KLE cells and by 34.9% in the Hec-1B cells, compared to untreated cells (Figure 2D, p<0.01). To better understand the effect of ascorbate on the mitochondrial apoptotic pathway, the KLE and Hec-1B cells were pretreated with a pan-caspase inhibitor Z-V-FMK (10 µM) for 2 hours, followed by treatment with ascorbate for 6 and 72 hours. ELISA results showed that Z-V-FMK pre-treatment effectively blocked the increase in levels of cleaved caspase 3 that were induced by ascorbate (1 mM) (Figure 2E, p<0.05). The MTT assay showed that Z-V-FMK significantly rescued the inhibition of cell proliferation by ascorbate (1 mM) in both cell lines, from 44.3% to 22.7% in KLE cells and from 18.5% to 1.3% in Hec-1B cells (Figure 2F, p<0.01). IHC results demonstrated that ascorbate reduced the expression of Bcl-xL by 41.2% and 35.8% in EC tumor tissues of obese and lean* LKB1^fl/fl^p53^fl/fl^-*mice, respectively, compared to control mice (Figure 2G, p<0.01). All these results indicate that inhibition of cell proliferation by ascorbate depends on the apoptotic pathway.

### Ascorbate inhibited adhesion and invasion in EC

**Figure 3:**
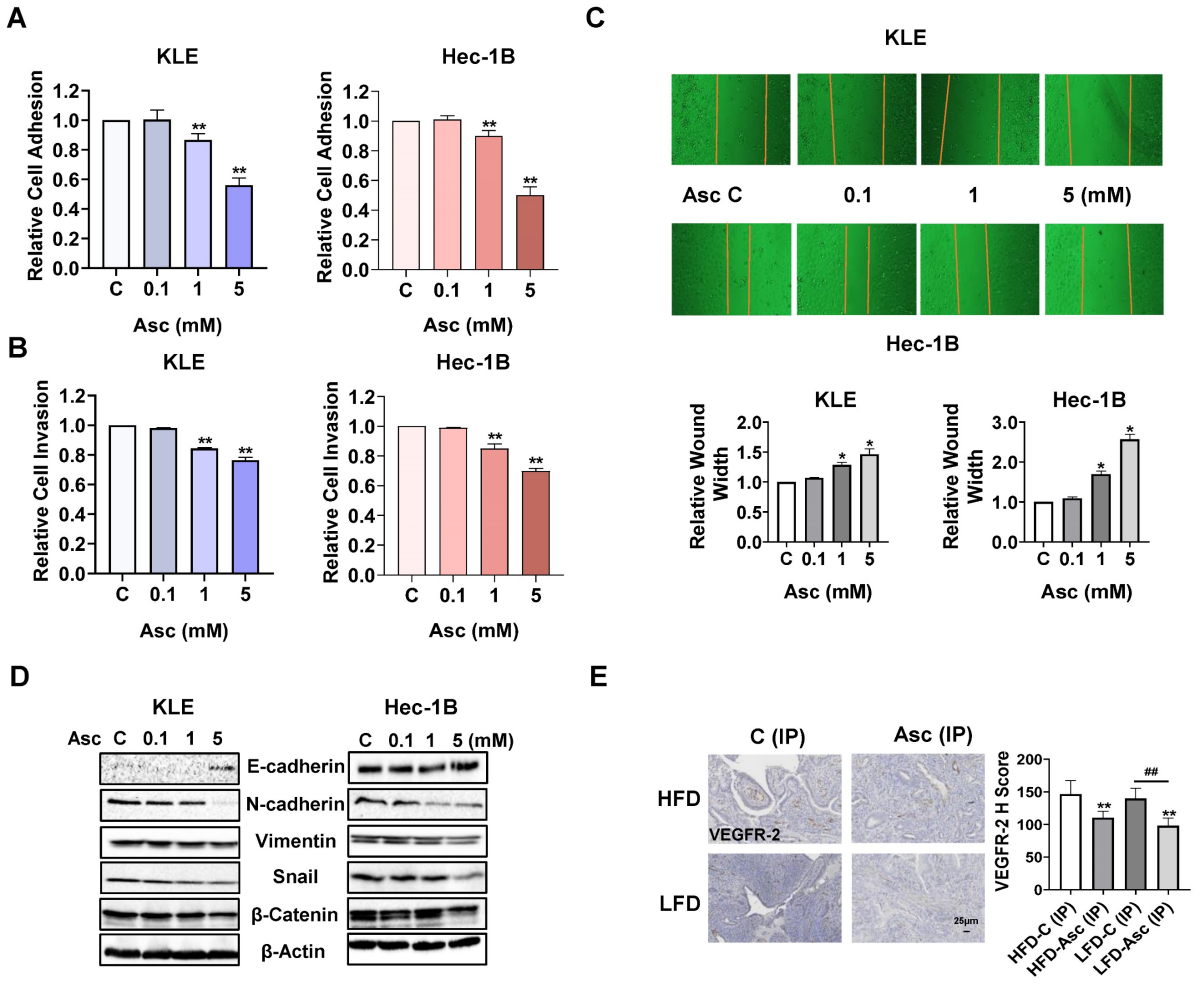
** Ascorbate inhibited adhesion and invasion in EC.** The KLE and Hec-1B cells were treated with 0.1, 1, and 5 mM ascorbate for 1.5 hours. The laminin-1 assay showed that 1 and 5 mM ascorbate significantly inhibited cell adhesion (A). The KLE and Hec-1B cells were treated with 0.1, 1, and 5 mM ascorbate for 2 - 4 hours. The transwell assay showed that 5 mM ascorbate inhibited cell invasion in both cells (B). The wound healing assay showed that 1 and 5 mM ascorbate suppressed cell migration after 24 hours of treatment in both cell lines (C). Western blotting showed that ascorbate impact the expression of E-cadherin, N-cadherin, Vimentin, snail, and β-catenin after 24 hours of treatment in both cell lines (D). IHC results demonstrated that ascorbate has the ability to reduce the expression of VEGFR-2 in endometrial tumor tissues of *LKB1^fl/fl^p53^fl/fl^*-mice in both the HFD and LFD groups (E). *p<0.05, **p<0.01 compared with C. *^#^p*<0.05, *^##^p*<0.01 compared with each group. Asc = Ascorbate, C (IP) = Vehicle control for IP injection group.

In order to investigate whether ascorbate reduces adhesion in EC, a laminin-1 adhesion assay was employed to determine adhesive ability in the KLE and Hec-1B cells. Ascorbate at doses of 1 and 5 mM significantly inhibited the cell adhesive ability of both cell lines, as 5 mM ascorbate inhibited adhesion by 43.9% and 49.9% in the KLE and Hec-1B cells, respectively, compared to the control cells (Figure 3A, p<0.01). Transwell assay also demonstrated that 1 and 5 mM ascorbate significantly inhibited the cell invasion ability in both cell lines. 5 mM ascorbate inhibited cell invasion by 23.6% and 30.2% in the KLE and Hec-1B cells, respectively, compared to the control cells (Figure 3B, p<0.01). The wound-healing assay further confirmed that ascorbate inhibited cell migration in both cell lines. 5 mM ascorbate increased wound width to 1.46-fold and 2.57-fold in the KLE and Hec-1B cells after 24 hours of treatment, respectively, compared to the control cells (Figure 3C, p<0.05). Considering epithelial-mesenchymal transition (EMT) is the initial step of tumor metastasis, the expression of proteins related to EMT was detected by Western blotting after the KLE and Hec-1B cells were exposed to 0.1, 1, and 5 mM ascorbate for 24 hours. Ascorbate increased the expression of E-cadherin and decreased the expression of N-cadherin, Vimentin, Snail, and β-catenin in a dose-dependent manner in both cell lines (Figure 3D). Vascular endothelial growth factor receptor 2 (VEGFR-2), the primary receptor mediating the effects of VEGF-A in cancer, is a key factor of the metastatic cascade, driving tumor angiogenesis, vascular permeability, lymphangiogenesis, and microenvironment remodeling 31-33. Hence, we detected the expression of VEGFR-2 in EC tissues of *LKB1^fl/fl^p53^fl/fl^-*mice under obese and lean conditions via IHC. Ascorbate inhibited the expression of VEGFR-2 by 24.6% and 29.8% in obese and lean groups, respectively, compared to the control mice (Figure 3E, p<0.01). These results indicate that ascorbate has the ability to inhibit tumor adhesion, migration, and invasion in EC *in vitro and in vivo*.

### Ascorbate induced cellular stress in EC

**Figure 4:**
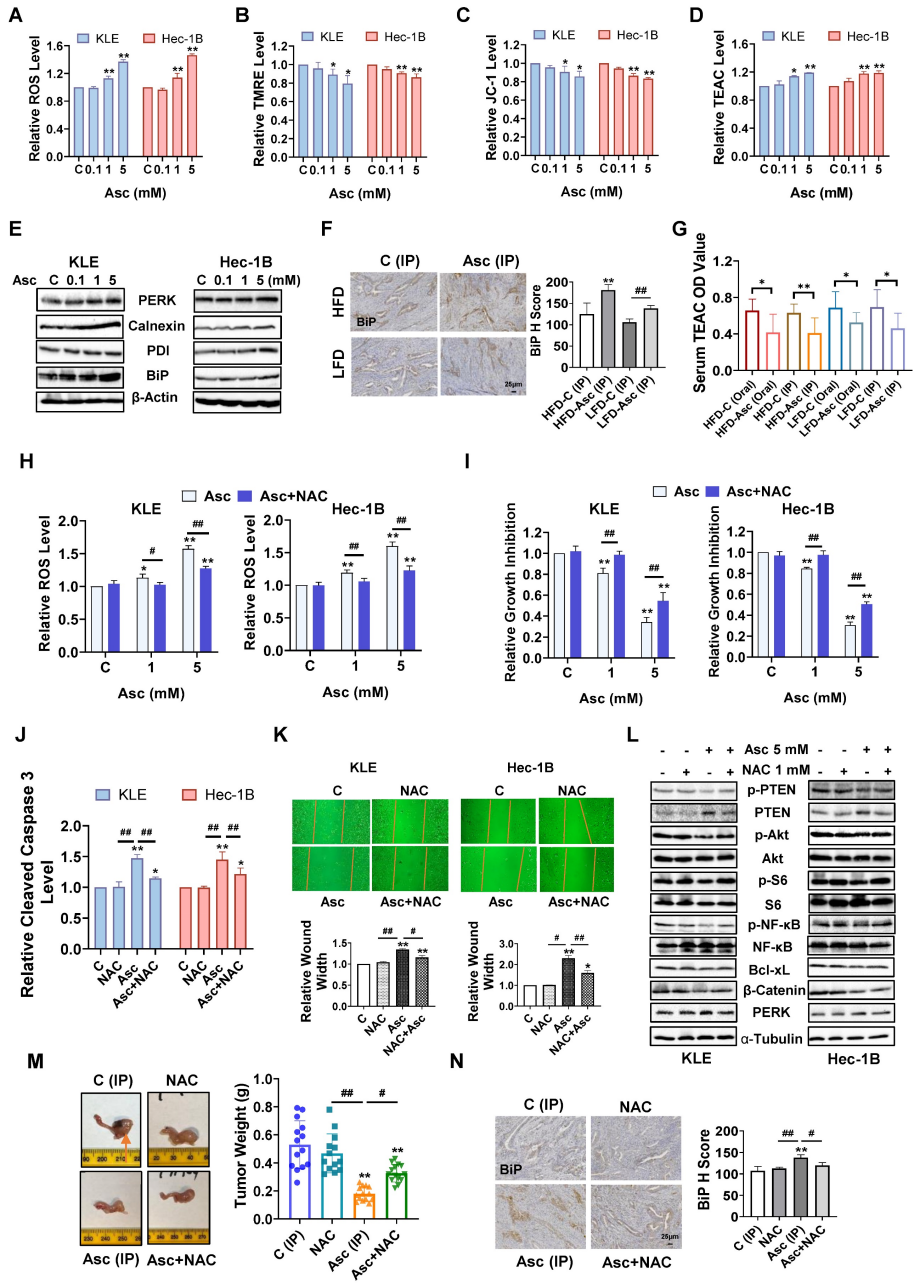
** Ascorbate induced cellular stress in EC.** Ascorbate treatment (6 hours) significantly increased the ROS levels and decreased the TMRE, and JC-1 levels in the KLE and Hec-1B cells (A, B, C). Ascorbate treatment (6 hours) significantly increased the TEAC levels in both cells (D). The effect of ascorbate treatment (6 hours) on the expression of PERK, Calnexin, PDI, and BiP in both cells (E). IHC demonstrated that ascorbate increased BiP expression in EC tissues of *LKB1^fl/fl^p53^fl/fl^*-mice in both HFD and LFD groups (F). Ascorbate administration (Oral or IP) reduced the TEAC level in both groups (G). After NAC (1 mM) pre-treated (3 hours), NAC totally reversed the increase of ROS levels induced by 1 mM ascorbate and partially reversed the increase of ROS induced by 5 mM ascorbate in both cells (H). MTT assay showed that NAC reversed the cell proliferation inhibition induced by 1 mM ascorbate and partially reversed the inhibition of proliferation induced by 5 mM ascorbate in both cells (I). The cleaved caspase 3 assay showed that NAC significantly eliminated the increase of cleaved caspase 3 induced by 5 mM ascorbate in both cells (J). The wound healing assay showed that NAC partially reversed the cell migration inhibition induced by ascorbate in both cells (K). The effect of NAC pre-treatment on the expression of Bcl-xL, β-catenin, PERK, PTEN, phosphorylated PTEN, phosphorylated AKT, phosphorylated S6, and phosphorylated NF-κB in ascorbate-treated cells in both cells (L). The lean *LKB1^fl/fl^p53^fl/fl^*-mice were treated with Ascorbate (IP), NAC, NAC+ Ascorbate (IP), or vehicle for four weeks, NAC combined with ascorbate reversed the inhibition of tumor weight induced by ascorbate (IP). The symbol (↑) indicates tumor (M). IHC results demonstrated that NAC reversed BiP elevation induced by ascorbate (IP) (N). *p<0.05, **p<0.01 compared with C. *^#^p*<0.05, *^##^p*<0.01 compared with each group. Asc = Ascorbate, C (Oral) = Vehicle control for oral administration group, C (IP) = Vehicle control for IP injection group.

High-dose ascorbate functions as a pro-oxidant compound to produce H_2_O_2_ in the presence of catalytic metal ions, triggering a significant increase in cellular stress 34, 35. To investigate the effect of ascorbate on cellular stress in EC, the KLE and Hec-1 cells were treated with 0.1, 1, and 5 mM ascorbate for 6 hours. Ascorbate, at doses of 1 and 5 mM, significantly increased cellular ROS production in the KLE and Hec-1B cells. 5 mM ascorbate increased the level of ROS by 37.0% in the KLE cells and by 46.4% in the Hec-1B cells, respectively, compared to the control cells (Figure 4A, p<0.01). The effect of ascorbate on mitochondrial membrane potential was detected by TMRE and JC-1 assays. Ascorbate at doses of 1 and 5 mM significantly decreased the mitochondrial membrane potential in both cells after 6 hours of treatment. Ascorbate (5 mM) significantly reduced TMRE levels by 20.6% and JC-1 levels by 14.3% in the KLE cells, and by 13.8 % and 16.7% in the Hec-1B cells (Figure 4B, C, p<0.05). Next, the TEAC assay was used to detect the total antioxidant capacity of KLE and Hec-1B cells after treatment with ascorbate for 6 hours. The higher OD value represents a lower antioxidant capacity. The results showed that 5 mM ascorbate significantly reduced the antioxidant capacity by increasing the TEAC level by 19.1% in the KLE cells and by 18.7% in the Hec-1B cells (Figure 4D, p<0.05). Western blotting showed that ascorbate increased the expression of the cellular stress-related proteins PERK, Calnexin, PDI, and BiP after 6 hours of treatment in the KLE and Hec-1B cell lines (Figure 4E).

In mouse studies, the IHC further demonstrated that ascorbate (IP) induced the expression of BiP by 44.9% and 29.0% in obese and lean groups, respectively, compared to control mice (Figure 4F, p<0.01). The antioxidant capacity of the serum of *LKB1^fl/fl^p53^fl/fl^-*mice was significantly increased after 4 weeks of treatment compared with control mice. Both oral and IP administrations of ascorbate reduced the plasma TEAC level in obese and lean mice, suggesting that ascorbate administration exerts an antioxidant and protective role in normal tissues where it gets replenished from plasma (Figure 4G, p<0.05).

Given that ascorbate exhibits pro-oxidant activity in EC cells, the antioxidant NAC was used to block ascorbate-induced cellular stress to observe whether blocking the cellular stress pathway would affect the inhibitory function of ascorbate. The KLE and Hec-1B cells were pre-treated with 1 mM NAC for 3 hours, and the results showed that NAC fully reversed the increase in ROS levels induced by 1 mM ascorbate and partially reversed the increase of ROS induced by 5 mM ascorbate in both cells. NAC reduced the increase of ROS level induced by 5 mM ascorbate from 57.3% to 27.4% in the KLE cells and from 59.9% to 22.6% in the Hec-1B cells (Figure 4H, p<0.01). The MTT assay showed that NAC reversed the inhibition of cell proliferation induced by 1 mM ascorbate in both cell lines and partially reversed the inhibition of proliferation induced by 5 mM ascorbate, from 65.6% to 45.1% in KLE cells and from 69.7% to 49.5% in Hec-1B cells (Figure 4I, p<0.01). A reduction of cleaved caspase 3 activities induced by ascorbate (5 mM) was observed after NAC pre-treatment for 3 hours, from 47.5% to 14.7% in KLE cells and from 45.1% to 21.8% in Hec-1B cells (Figure 4J, p<0.01). Furthermore, pre-treatment with NAC also partially reversed the cell migration inhibition induced by ascorbate (5 mM) in both cell lines, as the increase of wound width was reduced from 34.4% to 15.4% in the KLE cells and from 129.4% to 59.6% in the Hec-1B cells (Figure 4K, p<0.05). Western blotting confirmed that NAC increased the expression of Bcl-xL and β-catenin which was reduced by ascorbate and decreased the expression of PERK induced by ascorbate in both cells after 6 hours of treatment. NAC also reversed the ascorbate-driven induction of expression of PTEN and recovered the expression of phosphorylated PTEN, phosphorylated AKT, phosphorylated S6, and phosphorylated NF-κB that had been reduced by ascorbate in both cells after 24 hours of treatment (Figure 4L).

To better delineate the pro-oxidant role of ascorbate *in vivo*, the lean *LKB1^fl/fl^p53^fl/fl^-*mice were divided into: Control (IP) group, NAC group, ascorbate (IP) group, ascorbate (IP) + NAC group, and treated for 4 weeks. The administration of NAC alone did not cause significant changes in the body weight (Supplementary Figure 1C) or the tumor weight of mice (Figure 4M). The addition of NAC partially abolished the ascorbate (IP)-induced inhibition of tumor weight from 66.2% to 37.8% (Figure 4M, p<0.05). IHC results further confirmed that the BiP expression induced by ascorbate (IP) was reduced from 25.8% to 11.8% after the addition of NAC, indicating that an increase of cell stress at least partially underlined ascorbate-induced tumor growth inhibition (Figure 4N, p<0.05). Overall, both *in vitro and in vivo* results indicated that ascorbate exerts anti-tumorigenic and anti-invasive effects that are at least in part reliant on its pro-oxidant effect in EC.

### PTEN regulates the antitumor role of ascorbate in EC

**Figure 5:**
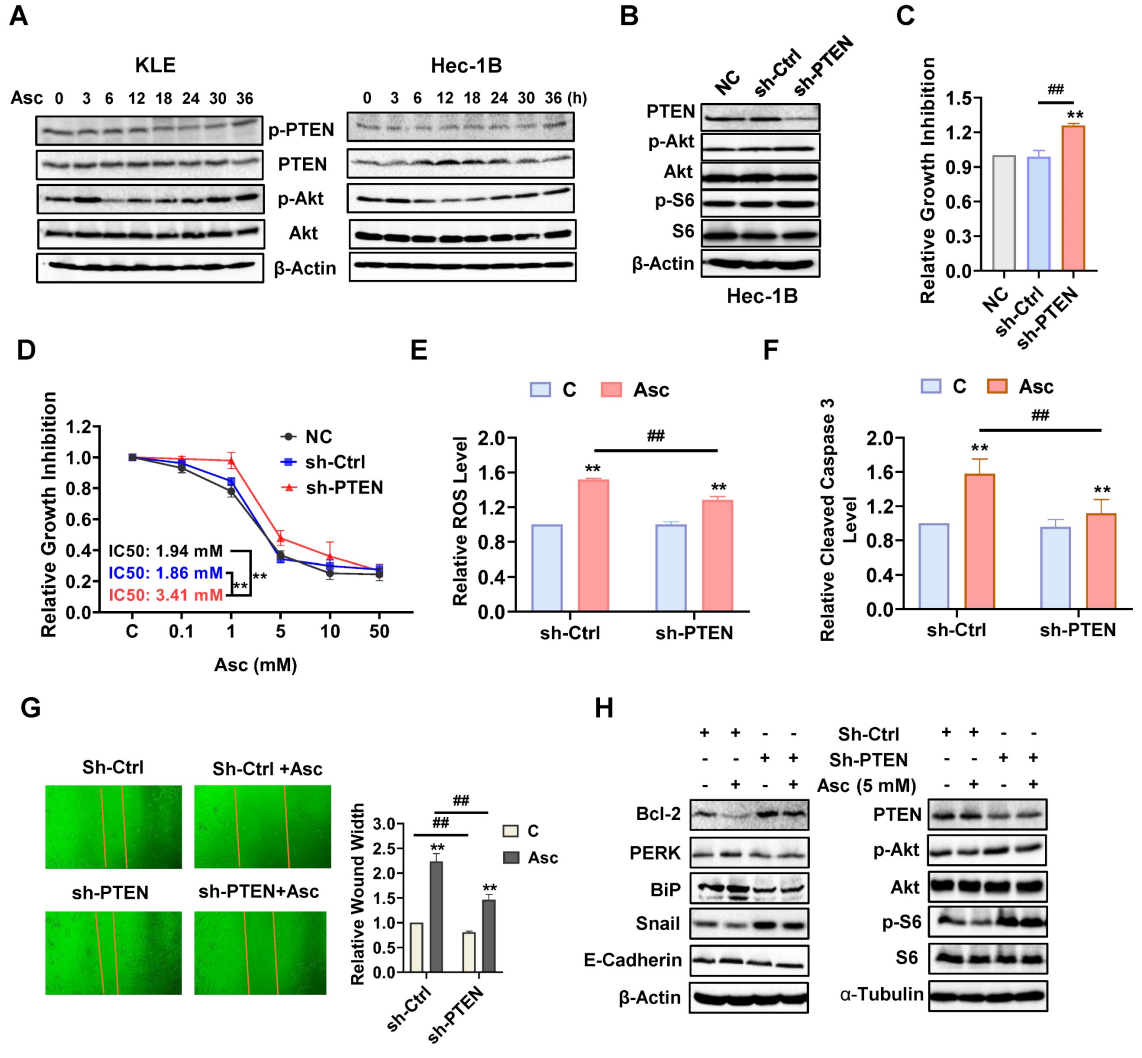
** PTEN regulates the anti-tumor role of ascorbate in EC cells.** The KLE and Hec-1B cells were treated with 5 mM ascorbate for 0, 3, 6, 12, 18, 24, 30, and 36 hours. Western blotting results showed the changes of PTEN, phosphorylated AKT, and phosphorylated S6 over time after ascorbate treatment in both cell lines (A). Sh-RNA was used to knock down the expression of PTEN in the Hec-1B cells. Sh-PTEN significantly decreased the level of PTEN and increased the expression of phosphorylated AKT and S6 in the Hec-1B cells compared to non-transfected and scramble control cells (B). MTT assay showed the inhibition of PTEN can induce cell proliferation and increase the IC50 of ascorbate in the EC cell lines (C, D). Downregulation of PTEN resulted in a decrease in the ROS level and cleaved caspase 3 activity induced by 5 mM ascorbate in sh-PTEN cells compared to scramble control cells after 6 hours of treatment (E, F). The wound healing assay showed that the inhibition of PTEN significantly accelerated cell migration in sh-PTEN cells and also weakened the cell migration inhibition induced by ascorbate in sh-PTEN cells compared to scramble controls after 24 hours of treatment (G). Western blotting showed that PTEN knockdown partially reversed the stimulatory effects of ascorbate on the expression of Bcl-2, PERK, BiP, Snail, E-cadherin, PTEN, phosphorylated AKT, and phosphorylated S6 (H). *p<0.05, **p<0.01 compared with scramble control or sh-PTEN control cells. *^#^p*<0.05, *^##^p*<0.01 compared with each group. Asc = Ascorbate.

Because ascorbate significantly increases the expression of wild-type PTEN and decreases the expression of phosphorylated PTEN in the KLE and Hec-1B cells, we further explored the role of the loss of PTEN in the regulation of ascorbate-driven inhibition of cell proliferation. The KLE and Hec-1B cells (both wild-type PTEN) were treated with 5 mM ascorbate in a time-course manner for 36 hours. Western blotting showed that ascorbate induced the expression of PTEN and decreased the expression of phosphorylated PTEN and phosphorylated AKT after treatment of 6 hours. Their expression levels in both cells gradually recovered over 24 hours of exposure (Figure 5A). Transfection of sh-PTEN into Hec-1B cells inhibited the expression of PTEN and increased the expression of phosphorylated AKT and phosphorylated S6 compared to non-transfected control (NC) and scramble control (sh-Ctrl) (Figure 5B). Knockdown of PTEN effectively facilitated cell proliferation after 72 hours of culture and decreased the sensitivity to ascorbate after 72 hours of treatment compared with control cells (Figure 5C and D, p<0.01). Treatment of sh-PTEN cells with 5 mM ascorbate significantly decreased the response to ascorbate-induced cellular stress compared with the control cells (Figure 5E, p<0.01). Similarly, PTEN downregulation attenuated the ability of ascorbate (5 mM) to induce cleaved caspase 3, with only an 11.8% increase in sh-PTEN cells compared to a 57.9% increase in sh-Ctrl after 6 hours of treatment (Figure 5F, p<0.01). To investigate the inhibitory effects of ascorbate on cell migration, sh-Ctrl cells and sh-PTEN cells were treated with ascorbate (5 mM) for 24 hours. Results showed that PTEN knockdown significantly facilitated cell migration in sh-PTEN cells compared to sh-Ctrl cells. Furthermore, the suppression of PTEN partially reversed the cell migration inhibition induced by ascorbate in sh-PTEN cells compared to sh-Ctrl cells, as ascorbate increased the wound width by 123.6% in sh-Ctrl cells compared to an 82.4% increase in sh-PTEN cells (Figure 5G, p<0.01). Moreover, Western blotting showed that PTEN knockdown partially reversed the stimulatory effect of ascorbate on the expression of Bcl-2, PERK, BiP, Snail, E-cadherin, PTEN, phosphorylated AKT, and phosphorylated S6 (Figure 5H). All of this combined suggests that PTEN regulates the inhibitory effect of ascorbate.

### Effect of targeting AKT on ascorbate-induced cell growth in EC

**Figure 6:**
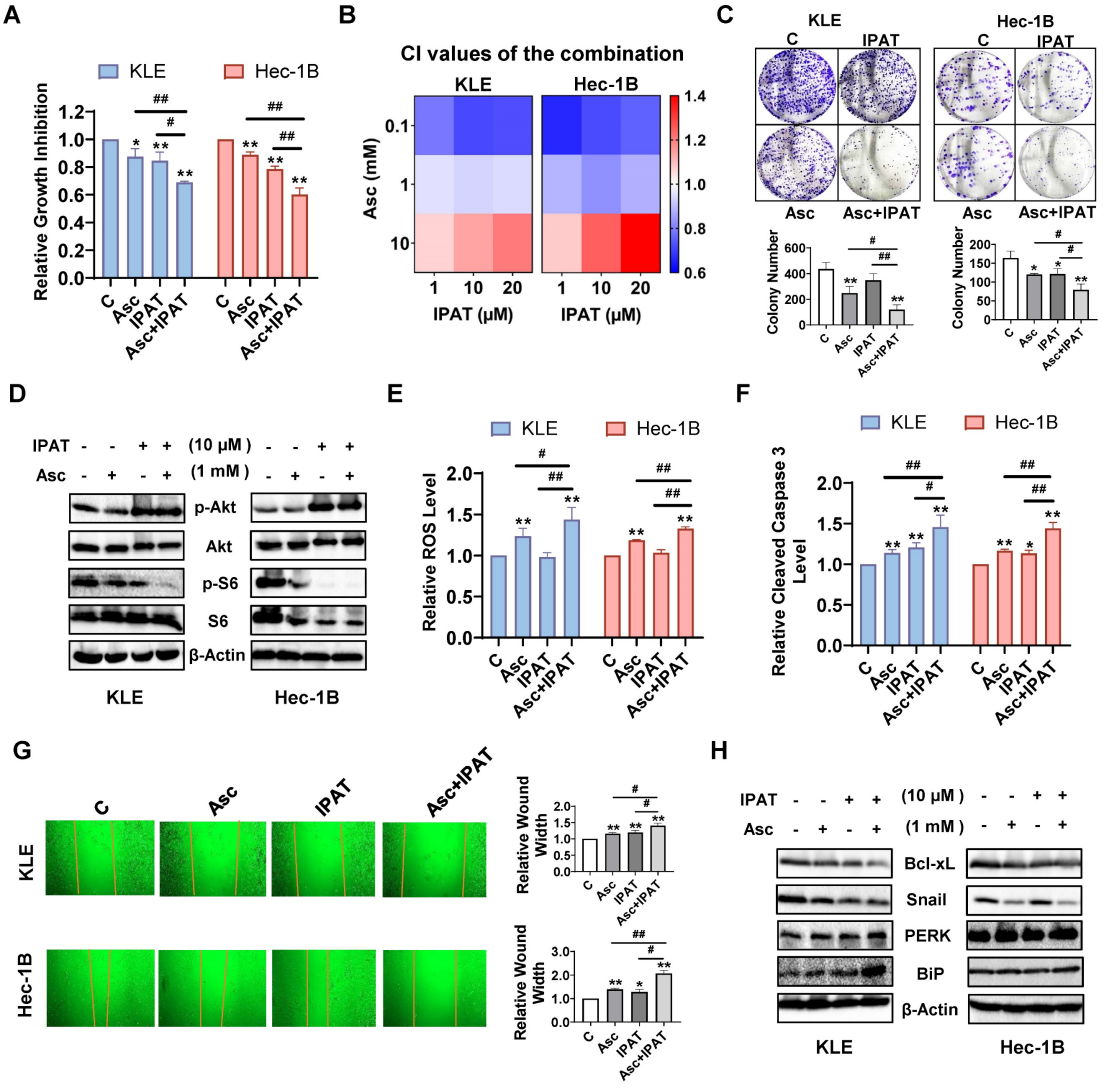
** Effect of targeting AKT on ascorbate-induced cell growth in EC cells.** The KLE and the Hec-1B cells were treated with 10 µM IPAT, 1 mM ascorbate, and the combination for 72 hours. MTT showed that the combination produced the most potent inhibitory effect on cell proliferation compared to IPAT or ascorbate alone in both cell lines (A). Both cells were treated with ascorbate (0.1, 1, and 10 mM), IPAT (1, 10, and 20 µM) for 72 hours. The synergy of ascorbate and IPAT was calculated based on the Bliss independence model (B). The colony formation assay also demonstrated consistent results in both cell lines after treatment with 10 µM IPAT, 1 mM ascorbate, and the combination for 24 hours (C). Western blotting results confirmed that IPAT (10 μM) significantly increased the expression of phosphorylated AKT and inhibited the expression of phosphorylated S6 in both KLE and Hec-1B cells. The combination of IPAT (10 μM) and ascorbate (1 mM) showed the lowest expression of phosphorylated S6 in both cell lines (D). The KLE and the Hec-1B cells were treated with 10 µM IPAT, 1 mM ascorbate, and the combination for 6 hours. The combination resulted in the strongest effects on increasing ROS levels and increasing cleaved caspase 3 levels in both cell lines compared to IPAT or ascorbate alone (E, F). The wound healing assay showed the highest cell migration inhibitory effects in the combination groups in both cell lines after 24 hours of treatment (G). Western blotting results showed that the combination had a stronger inhibitory effect on the expression of Bcl-xL and Snail and demonstrated the highest elevation in the expression of BiP and PERK compared to each drug alone. (H). *p<0.05, **p<0.01 compared with C. *^#^p*<0.05, *^##^p*<0.01 compared with each group. Asc = Ascorbate.

Since PTEN loss resulted in increased expression of phosphorylated AKT and phosphorylated S6, we next investigated the effect of the AKT/mTOR pathway on the inhibition of cell proliferation by ascorbate. The KLE and Hec-1B cells were treated with 1mM ascorbate, 10 μM IPAT (a potent AKT inhibitor), or the combination of ascorbate and IPAT for 72 hours. MTT assay showed that either 1mM ascorbate or 10 μM IPAT significantly reduced cell proliferation, whereas the combination treatment produced a more potent inhibitory effect in both cell lines (Figure 6A, p<0.05). To further determine whether the combination treatment produces a synergistic effect, both cell lines were treated with different concentrations of ascorbate, IPAT, and the combination for 72 hours. The CI of each combination group was calculated using the Bliss independence model based on the MTT results. The combination of ascorbate (0.1 and 1mM) and IPAT (1, 10, and 20 μM) showed significant synergy in inhibiting cell proliferation (Figure 6B, CI < 1). The colony formation assay confirmed the combination of ascorbate (1 mM) and IPAT (10 μM) inhibited more colony formation compared with IPAT alone, ascorbate alone, and the control cells in both cell lines (Figure 6C, p<0.05). Western blotting results found that the combination of IPAT (10 μM) and ascorbate (1 mM) produced a more obvious inhibitory effect on the phosphorylation of S6 in both cell lines, suggesting that the AKT/mTOR pathway is involved in the inhibitory function of ascorbate on cell proliferation (Figure 6D). Furthermore, the combination groups demonstrated the highest increase in ROS levels compared to each drug alone, as the combination group increased ROS level by 44.4% compared to 23.2% with ascorbate alone and no effect with IPAT alone, respectively, in KLE cells, and increased ROS level by 33.1% compared to 17.9% of ascorbate alone and no effect with IPAT alone, respectively, in Hec-1B cells (Figure 6E, p<0.05). Cleaved caspase 3 activity was also significantly increased by 46.2% in KLE cells and 43.7% in Hec-1B cells after being exposed to the combination treatment for 6 hours, compared to 14.1% and 20.5% of ascorbate and IPAT alone in KLE cells, respectively and 16.4% and 13.1% of ascorbate and IPAT alone in Hec-1B cells, respectively (Figure 6F, p<0.01). As for cell migration, the combination group caused a 40.4% increase in wound width in KLE cells, compared to 16.1% with ascorbate alone and 18.8% with IPAT alone. Similarly, in Hec-1B cells, the wound width increased by 107.7% with the combination treatment, compared to 39.2% with ascorbate alone and 27.8% with IPAT alone (Figure 6G, p<0.05). Western blotting showed that the combination of ascorbate and IPAT had a stronger inhibitory effect on the expression of Bcl-xL and Snail and a more potent effect on increasing the expression of BiP and PERK compared to either drug alone and the control group (Figure 6H). Collectively, these data suggest that the PTEN/AKT/mTOR pathway is critical to the anti-tumorigenic activities of ascorbate by influencing cell proliferation, apoptosis, cell stress, and migration in EC cells.

### Ascorbate in combination with paclitaxel synergistically inhibits cell proliferation and tumor growth in EC

**Figure 7:**
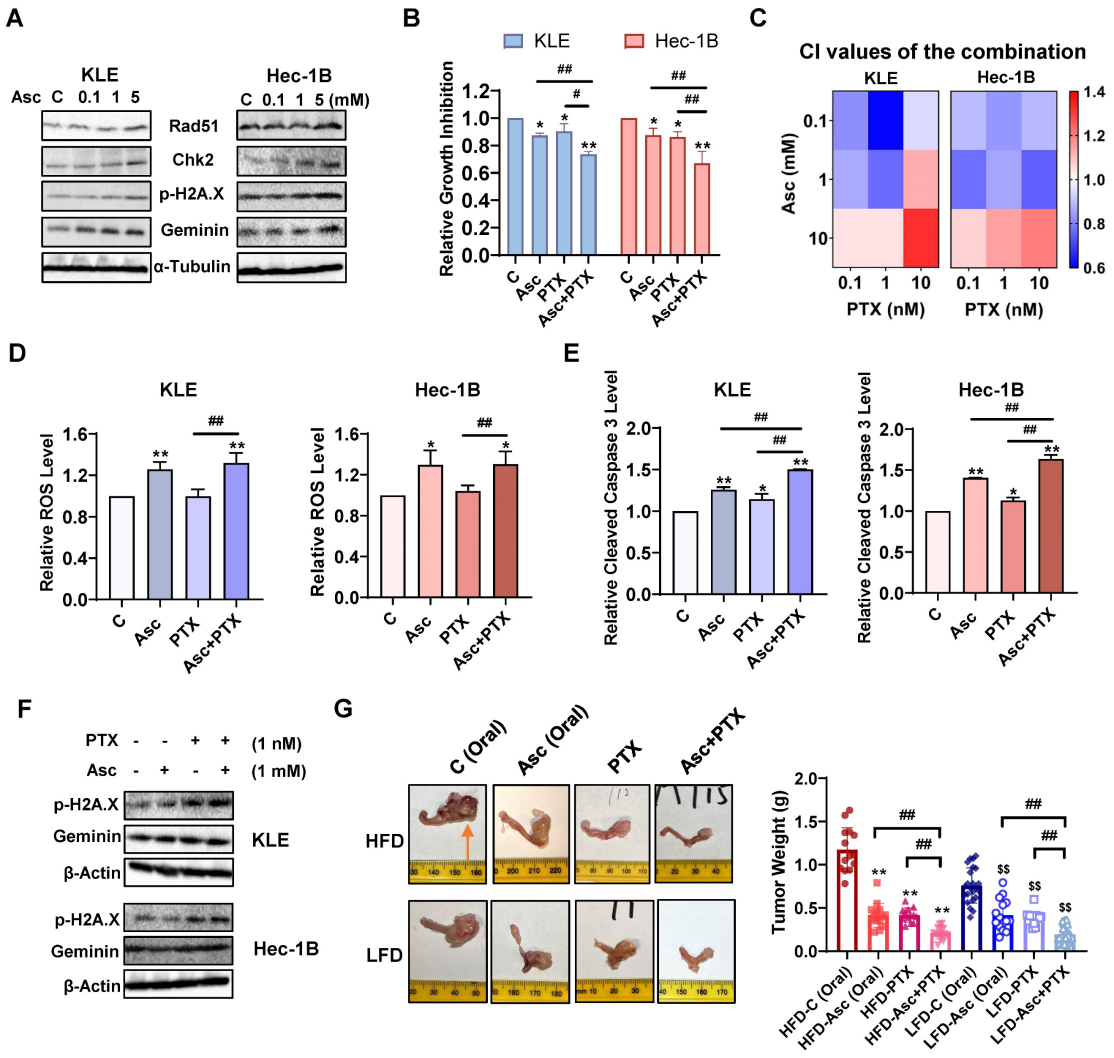
** Ascorbate in combination with paclitaxel synergistically inhibits cell proliferation and tumor growth in EC.** Western blotting results showed that ascorbate enhanced the expression of Rad51, Chk2, p-H2A.X, and Geminin in both cells after 6 hours of treatment (A). The combination of ascorbate (1 mM) and PTX (1 nM) resulted in the greatest cell growth inhibition than ascorbate or PTX alone in both cell lines after 72 hours of treatment (B). The KLE and the Hec-1B cells were treated with ascorbate (0.1, 1, and 10 mM), PTX (0.1, 1, and 10 nM), and combination for 72 hours. The synergy of ascorbate and PTX was calculated based on the CI value by the Bliss independence model (C). The combination of ascorbate (1 mM) and PTX (1 nM) was used in the following studies. The combination significantly increased ROS levels compared to PTX alone after treatment for 6 hours in both cell lines (D). The combination demonstrated the highest cleaved caspase 3 levels than each drug alone after 6 hours of treatment in both cell lines (E). Western blotting showed the combination exerted a more potent effect in the expression of phosphorylated H2A.X and Geminin compared to each drug alone after 6 hours of treatment in both cell lines (F). In order to confirm the synergistic effect of ascorbate and PTX *in vivo*, the obese and lean *LKB1^fl/fl^p53^fl/fl^*-mice were divided into 8 groups. The combination group demonstrated the highest tumor growth inhibition in comparison with the control groups in both obese and lean mice. The symbol (↑) indicates tumor (G). *p<0.05, **p<0.01 compared with Control *in vitro* and compared with HFD-C *in vivo*. *^$^p*<0.05, *^$$^p*<0.01 compared with LFD-C *in vivo*. *^#^p*<0.05,*^ ##^p*<0.01 compared with each group. Asc = Ascorbate, C (Oral) = Vehicle control for oral administration group.

Ascorbate has been reported to induce DNA damage in various types of cancer, including both ovarian and pancreatic cancer 24, 36. To investigate whether ascorbate induces DNA damage in EC cells, the KLE and Hec-1B cells were treated with 0.1, 1, and 5 mM ascorbate for 6 hours, and Western blotting results confirmed that ascorbate significantly increased the protein expression of Rad51, Chk2, phosphorylated H2A.X, and Geminin in both cells (Figure 7A). Given that PTX is a DNA damage inducer and the PTEN/AKT/mTOR pathway is associated with its anti-proliferative (pro-apoptotic) role in cancer cells, the synergistic effect of ascorbate and PTX on cell proliferation in KLE and Hec-1B cells was evaluated by MTT assay. Treatment with 1 mM ascorbate and 1 nM PTX led to 26.4% and 33.1% cell growth inhibition in KLE and Hec-1B cells respectively, whereas ascorbate and PTX alone produced 12.8% and 9.8% cell growth inhibition in KLE cells, and 12.5% and 13.8% in Hec-1B cells, respectively (Figure 7B, p<0.01). After treatment with three doses of ascorbate and PTX for 72 hours in both cell lines, the combination of ascorbate (0.1, 1 mM) and PTX (0.1, 1, and 10 nM) exhibited potent synergy in the inhibition of cell proliferation in both KLE and Hec-1B cells (Figure 7C, CI <1). Although the combination of ascorbate and PTX did not result in an increase in ROS levels compared to ascorbate alone after 6 hours of treatment in both cell lines, this combination significantly increased the ROS levels compared to PTX (Figure 7D, p<0.05). The combination also significantly increased cleaved caspase 3 activity by 50% and 64% in KLE and Hec-1B cells respectively, compared to each drug alone (Figure 7E, p<0.05). Additionally, Western blotting showed that the combination of ascorbate and PTX exerted a more potent effect on the expression of DNA damage-marker - phosphorylated H2A.X and Geminin - compared to ascorbate or PTX alone after 6 hours of treatment in both cell lines (Figure 7F). These results suggest that ascorbate may enhance synergy with PTX by inducing ROS, apoptosis, and DNA damage in EC cells.

To investigate the synergistic effect of ascorbate and PTX *in vivo*, the *LKB1^fl/fl^p53^fl/fl^*-mice under obese and lean conditions were divided into 8 groups in total: HFD-vehicle (Oral), HFD-Ascorbate (Oral), HFD-PTX, HFD-Ascorbate (Oral) + PTX; LFD-vehicle (Oral), LFD-Ascorbate (Oral), LFD-PTX, LFD-Ascorbate (Oral) + PTX. Following four weeks of treatment, tumor weights were significantly reduced in all treatment groups, with the combination group demonstrating the highest inhibition rate in comparison with the control groups in both obese and lean mice. In obese mice, the combination of ascorbate and PTX inhibited tumor weight by 82.1% compared to 64.5% and 64.6% of PTX and ascorbate alone, respectively. The lean mice also demonstrated similar results as the combination led to a 75.9% inhibition in comparison with 49.3% and 44.1% of PTX and ascorbate alone, respectively (Figure 7G, p<0.01). Besides, compared with single agents, the combination had no impact on the body weight of mice and did not cause any changes in cell morphology and tissue architecture of major organs such as the heart, liver, spleen, lungs, and kidneys (Supplementary Figure 1A and D). These results confirmed that the combination treatment of ascorbate and PTX exhibits synergistic effects in cell proliferation and tumor growth *in vitro and in vivo* in EC.

## Discussion

Ascorbate, a classic bioactive compound, has received attention in recent years for its potential anti-tumorigenic activity. New evidence has emerged to confirm that millimolar level of ascorbate in extracellular fluid exerts potent anti-tumorigenic activity in pre-clinical studies and exhibits favorable therapeutic responses and good safety profiles in cancer patients in several clinical trials 8, 15. In this study, we investigated the effect of ascorbate on cell proliferation in EC cell lines and tumor growth in the *LKB1^fl/fl^p53 ^fl/fl^*-transgenic mouse model of EC. Our results confirmed that ascorbate at millimolar levels significantly inhibited cell proliferation, induced cell cycle arrest, autophagy, ROS, DNA damage, and apoptosis, suppressed cell adhesion, invasion, and migration, and decreased EC tumor growth under obese and lean conditions. In a dose-dependent manner, ascorbate increased the expression of wild-type PTEN and decreased phosphorylation of AKT and S6 in the KLE and Hec-1B cells. Knockdown of PTEN or inhibition of the AKT/mTOR pathway by IPAT effectively affected the sensitivity of KLE and Hec-1B cells to ascorbate, suggesting that anti-tumorigenic activity of ascorbate is at least in part dependent on the PTEN/AKT/mTOR pathway. More importantly, ascorbate in combination with PTX demonstrated potent anti-tumorigenic effects compared to either drug alone in EC cells and *LKB1^fl/fl^p53^fl/fl^*-transgenic mice, providing promising pre-clinical evidence of the potential combination of ascorbate and PTX in EC treatment.

Physiological concentrations of ascorbate are involved in various physiological processes in normal cells through its antioxidant effects, while pharmacological doses of ascorbate exhibit anti-tumorigenic activity through its pro-oxidative activity. The oxidative stress in cancer cells and tumors was induced via the superoxide (or metal ion)-driven formation of ascorbate radical (Asc•-) with subsequent production of H_2_O_2_ 37-40. The anti-tumorigenic activity of high-dose ascorbate generally depends on the production of H_2_O_2_ in the extracellular fluid of tumor-bearing animals and is linearly related to the formation of ascorbyl radicals 37, 41. Normal tissues contain higher amounts of antioxidant enzymes, in particular H_2_O_2_ - scavenging enzymes when compared to cancer cells, making it improbable that elevated levels of H_2_O_2_ would cause harm to normal cells 20, 22. Increased oxidative stress has been shown to influence translation, cell cycle profile, apoptosis, adhesion, protein folding, and autophagy through oxidation of large amounts of peptidyl cysteines in cancer cells 16. Our study confirmed that millimolar levels of ascorbate led to increased ROS production, accompanied by induction of apoptosis, cell cycle G1 arrest and DNA damage, and reduced cell invasion in EC cells. Pre-treatment of EC cells with 1 mM NAC fully reversed the 1 mM ascorbate-induced inhibition of ROS production and cell proliferation and partially reversed the 5 mM ascorbate-induced enhancing of ROS levels, cell migration, and cleaved caspase levels, and blunting of cell viability. In addition, NAC partially abolished tumor growth inhibition induced by ascorbate (IP) in our *LKB1^fl/fl^p53^fl/fl^*-mice. Consistent with our results, some studies also found that NAC completely reversed cell and tumor growth inhibition induced by 1-2 mM ascorbate in thyroid cancer and colorectal cancer, and partially reversed the inhibition induced by 3 and 10 mM ascorbate in breast cancer 42-44. These results suggest that NAC may be insufficient in blocking ROS production induced by 5 mM ascorbate or that ascorbate may have another underlying mechanism involved in its anti-tumorigenic activity, in addition to its pro-oxidative effects. In support of this, we pre-treated KLE cells with the pan-caspase inhibitor Z-V-FMK and found that Z-V-FMK could not completely reverse the cell growth inhibition induced by 1 mM ascorbate, suggesting that ascorbate may have multiple mechanisms by which it blunts cell growth.

The inter-relationship of different administration routes and the tumor growth inhibitory effects of ascorbate have been widely studied in numerous cancer models. Oral administration of ascorbate, limited by the small intestine, cannot achieve millimolar levels of bodily extracellular fluids, resulting in inconsistent anti-tumorigenic effects in varying pre-clinical cancer models 45. The concentration of ascorbate in drinking water at 3.3 g/L significantly increased the concentration of ascorbate in plasma and tumor tissues and showed a greater tumor growth inhibition compared with the ascorbate concentrations of 0.33 g/L and 0.033 g/L in melanoma and lung carcinoma mouse models 46, 47. In methylcholanthrene (MCA)-induced cervical cancer and soft tissue sarcoma mouse models, ascorbate (2-5 g/L) in drinking water were found to have a tumor-preventive effect, but in another soft tissue sarcoma mouse model, these same levels of ascorbate failed to demonstrate any significant anti-tumor properties 48, 49. Recent studies have found that the concentration of ascorbate in extracellular fluid is key to maintaining the anti-tumorigenic activity of ascorbate. IP injection of ascorbate (1 g/kg) rapidly increases plasma ascorbate to over 15 mM, but the half-life of ascorbate in the blood is only about 77 minutes in mice 37, 38, 41. Surprisingly, ascorbate levels in the extracellular fluid surrounding tumors can remain elevated for more than 24 hours due to the increased stability in the hypoxic tumor environment (rich in oxygen-derived free radicals such as superoxide) and the poor vascularization-induced delayed clearance, which results in H_2_O_2_ accumulating to higher concentrations in the extracellular space around tumors, ultimately becoming toxic to cancer cells 14, 41. It has been confirmed that IP injection of high-dose ascorbate (1-4 g/kg, once or twice a day) can successfully inhibit tumor growth in over 10 types of cancer without obvious toxicity and may even show protective antioxidant effects on normal tissues 15. In humans, a linear relationship between ascorbate dose and peak plasma concentration has been observed for doses up to approximately 70 g/m², leading to a plasma concentration of around 50 mM 14. The 1.5 g/kg (intravenous) ascorbate commonly used in clinical trials is sufficient to maintain plasma ascorbate concentrations > 10 mM for several hours without causing significant side effects 50. In this study, we first compared the anti-tumorigenic effects of two administration routes in the *LKB1^fl/fl^ p53^fl/fl^*-transgenic mouse model of EC. High-dose ascorbate administered orally and intraperitoneally showed significant inhibition of tumor growth in both obese and lean conditions. However, obese mice were more sensitive to ascorbate treatment regardless of the route of administration. Consistent with other studies, IP injection of high-dose ascorbate was more effective than oral administration in inhibiting tumor growth in obese and lean mice, indicating the importance of the administration route and the achievement of millimolar level in the application of ascorbate in the treatment of patients.

The mutation and deletion of tumor suppressor gene PTEN accompanied by activation of the PI3K/AKT/mTOR signaling is one of the most common molecular events in EC 3-5. The PI3K/AKT/mTOR pathway is involved in the inhibitory effect of ascorbate on cell proliferation and tumor growth. High-dose of ascorbate inhibited cell proliferation and tumor growth through ROS-dependent inhibition of the MAPK/ERK and PI3K/AKT pathways in thyroid cancer 42. In Ehrlich-induced breast cancer in mice, ascorbate (4 g/kg/day, IP) treatment for 15 days exerted anti-neoplastic activity through inhibition of the PI3K/AKT/mTOR pathway 51. In the current study, we found for the first time that ascorbate at millimolar levels significantly increased the expression of wild-type PTEN and decreased the expression of phosphorylated PTEN, AKT, and S6 in the KLE and Hec-1B cells. Ascorbate (IP) increased the expression of wild-type PTEN in EC tissues and the contralateral normal endometrium while decreasing the expression of phosphorylated S6 in EC tumor tissues. Pretreatment of both cells with NAC partially reversed the ascorbate-induced changes in the expression of phosphorylated PTEN, phosphorylated AKT, and phosphorylated S6, indicating that ascorbate regulates the PTEN/AKT pathway partially through its pro-oxidant effects. The knockdown of PTEN in Hec-1B cells by lentivirus reduced the sensitivity of cells to ascorbate and attenuated ascorbate-induced ROS, apoptosis, and migration. In contrast, targeting AKT by the AKT inhibitor IPAT effectively enhanced the effects of ascorbate on cell proliferation, cellular stress, apoptosis, and cell migration in the KLE and Hec-1B cells. These results confirm that ascorbate exhibits anti-tumorigenic activity, with these effects at least in part dependent on the PTEN/AKT/mTOR pathway in wild-type PTEN EC cells. Our data agree well with the impact of Mn porphyrin/radiation-induced H_2_O_2_ production on many of the cellular metabolic pathways studied herein and summarized in references 18, 22, and 23.

Ascorbate has also been shown to have anti-metastatic activity in a variety of different pre-clinical cancer models. The anti-metastatic activity of ascorbate has been associated with the regulation of several metastasis-related processes including EMT, extracellular matrix (ECM), and angiogenesis in animal models 52-55. Millimolar levels of ascorbate inhibited the expression of the EMT-related protein Snail, Vimentin, and N-cadherin expression in breast, pancreatic, and colon cancer cells and mouse models 52-54. Ascorbate, at the dose of 1 mM, inhibited ECM degradation and decreased the expression of metalloproteinases (MMPs) 2 and 9 via a hydrogen peroxide-mediated mechanism in pancreatic cancer cells. Furthermore, injection of ascorbate (4 g/kg, IP) for 30 days decreased the absolute number of circulating tumor cells and inhibited hepatic metastases in a xenograft mouse model of pancreatic cancer 56. Downregulation of VEGF expression in tumor tissues and decreased secretion of serum VEGF were found in xenograft mouse models of melanoma, breast cancer, and pancreatic cancer treated with ascorbate 57, 58. Moreover, ascorbate (1g/kg, IP) for 4 weeks significantly reduced micro-vessel density in a xenograft mouse model of lung cancer 41. Similarly, our current study confirmed that ascorbate suppressed cell adhesion, invasion, and migration and decreased the expression of EMT-related proteins such as Vimentin, Snail, and β-catenin in KLE and Hec-1B cells and that pre-treatment with NAC partially eliminated ascorbate-induced inhibition of cell migration. We also found that changes in the expression of PTEN and AKT had an impact on the inhibitory function of ascorbate in cell migration. Suppression of PTEN reduced the ability of ascorbate to inhibit cell migration, whereas inhibition of the AKT pathway effectively increased ascorbate's inhibitory effects on cell migration. Ascorbate (IP) treatment in *LKB1^fl/fl^ p53^fl/fl^*-mice effectively reduced the expression of VEGFR-2 in EC tumors compared to the control group. These data suggest that ascorbate-regulated inhibition of cell adhesion and invasion may be related to inhibition of the PTEN/AKT pathway.

The ability of ascorbate to induce ROS-dependent DNA damage has been confirmed in a variety of cancers. Ascorbate at millimolar levels significantly induces DNA damage through increasing high fluxes of H_2_O_2_ and activating Chk1 kinase and PARP1 pathways in pancreatic cancer cells 36. Treatment of non-small cell lung cancer cells with 6 mM ascorbate increased the expression of γ-H2AX, p-Chk1, and p-RPA2, and disrupted the accumulation of DNA repair factors at damaged sites via H_2_O_2_ production 59. Our results showed that 1 and 5 mM ascorbate significantly increased the expression of Rad51, Chk2, phosphorylated H2A.X, and Geminin in EC cells. Given that ascorbate-induced H_2_O_2_ combined with H_2_O_2_ from other sources can produce greater toxicity to cancer cells, we speculate that the combination of ascorbate and PTX may enhance anti-tumorigenic activity by enhancing DNA damaging effects in EC cells 19. Namely, ascorbate-mediated superoxide production may accelerate oxidative DNA damage by inducing iron release from storage proteins or enzymic [4Fe-4S] clusters, which then may deposit on the DNA surface, causing oxidative damage to DNA 60. Our experiments combining ascorbate and PTX treatments support the hypothesis, demonstrating synergistic effects through induction of ROS, cleaved caspase 3 activity, and DNA damage. Notably, the time-course analysis revealed 1 nM PTX increased ROS levels after 16 hours of treatment, while 1 mM ascorbate significantly elevated ROS levels within 1-12 hours and peaked at 6 hours. However, although combined treatment of EC cells with 1 nM PTX and 1 mM ascorbate for 6 hours did not increase ROS production compared with ascorbate alone, the combination treatment for 6 hours synergistically increased cleaved caspase 3 activity compared with treatment with PTX or ascorbate alone, suggesting that low-dose paclitaxel can effectively promote the pro-oxidative and apoptosis-inducing effects of ascorbate in EC cells. Employing the *LKB1^fl/fl^ p53 ^fl/fl^*-mice, we explored the effects of combining ascorbate and PTX on tumor growth *in vivo*. The combination of ascorbate and PTX led to the most significant inhibition of tumor growth in both obese and lean *LKB1^fl/fl^ p53 ^fl/fl^*-mice compared to either treatment alone. Furthermore, the combination treatment demonstrated great safety profiles without affecting the body weight and causing morphological changes in major organs in mice. These findings offer valuable pre-clinical rationale for the consideration of a clinical trial investigating the combination of ascorbate and PTX in the treatment of EC.

## Conclusions

Our study demonstrated that high-dose ascorbate inhibited EC cell proliferation, cell invasion, and tumor growth. We found that IP administration of ascorbate was more effective in the inhibition of tumor growth than oral administration. NAC reversed the anti-tumorigenic and anti-invasive effects of ascorbate, suggesting that ascorbate exerts its inhibitory effects partially via its function as a pro-oxidant. Ascorbate upregulated PTEN expression, and the knockdown of PTEN made EC cells less sensitive to ascorbate, while targeting AKT by IPAT increased sensitivity to ascorbate. These findings support that the anti-tumorigenic effects of ascorbate may be reliant on crosstalk with the PTEN/AKT pathway in EC. Additionally, ascorbate in combination with PTX demonstrated synergistic anti-tumorigenic effects in EC cells and the *LKB1^fl/fl^p53 ^fl/fl^*-transgenic mouse EC model. Most notably, we demonstrated for the first time that ascorbate impacts functional PTEN; thus, the underlying mechanism of how ascorbate regulates the PTEN/AKT signaling pathway in EC requires further exploration, especially given that this pathway is frequently altered in this disease. Lastly, this study suggests that ascorbate in combination with paclitaxel may be a promising combination therapy for EC treatment, a disease that has limited therapeutic options.

## Supplementary Material

Supplementary figures.

## Abbreviations

Ad-Cre: Ad5-CMV-Cre
BCA: Bicinchoninic acid
CI: Combination index
DAB: 3,3'-Diaminobenzidine
DCFH-DA: 2,7-dichlorodihydrofluorescein diacetate
DMSO: Dimethyl sulfoxide
EC: Endometrial cancer
ECM: Extracellular matrix
EMT: Epithelial-mesenchymal transition
FBS: Fetal bovine serum
H_2_O_2_: Hydrogen peroxide
H&E: Hematoxylin and Eosin
HFD: High-fat diet
HIF-1: Hypoxia-inducible factor-1
IACUC: Institutional Animal Care and Use Committee
IHC: Immunohistochemistry
IP: Intraperitoneal
IPAT: Ipatasertib
LFD: Low-fat diet
MCA: Methylcholanthrene
MMPs: Metalloproteinases
MnPs: Mn (III) ortho N-alkyl- and N-alkoxyalkyl porphyrins
NAC: N-acetylcysteine
PTX: Paclitaxel
ROS: Reactive oxygen species
SDS-PAGE: Sodium dodecyl sulfate-polyacrylamide gel electrophoresis
TEAC: Trolox equivalent antioxidant capacity
UNC-CH: University of North Carolina at Chapel Hill
VEGFR-2: Vascular endothelial growth factor receptor 2
Z-V-FMK: Z-VAD-FMK
