# Supplementary material for: High-dose Ascorbate Exhibits Anti-proliferative and Anti-invasive Effects Dependent on PTEN/AKT/mTOR Pathway in Endometrial Cancer in vitro and in vivo
Source: Int J Biol Sci. 2025 Jan 27;21(4):1545–65. doi: 10.7150/ijbs.102079 (PMC11844297; doi:10.7150/ijbs.102079)
Supplement: Supplementary file 1 — Supplementary figures. [file ijbsv21p1545s1.pdf]

High-dose Ascorbate Exhibits Anti-proliferative and Anti-invasive Effects Dependent on PTEN/AKT/mTOR Pathway in Endometrial Cancer *in vitro* and *in vivo*

Xiaochang Shen[1,2*], Jiandong Wang[1*], Weimin Kong[1], Catherine John[2], Boer Deng[1,2], Shuning Chen[1,2], Haomeng Zhang[1,2], Jennifer Haag[2], Nikita Sinha[2], Wenchuan Sun[2], Angeles Alvarez Secord[3], Chunxiao Zhou[2,4**], and Victoria L Bae-Jump[2,4**]

1. Department of Gynecological Oncology, Beijing Obstetrics and Gynecology Hospital, Capital Medical University, Beijing Maternal and Child Health Care Hospital, Beijing 100006, PR China.
2. Division of Gynecologic Oncology, Department of Obstetrics and Gynecology, University of North Carolina at Chapel Hill, Chapel Hill, NC 27599, USA.
3. Division of Gynecologic Oncology, Department of Obstetrics and Gynecology, Duke Cancer Institute, Duke University, Durham, NC 27710, USA.
4. Lineberger Comprehensive Cancer Center, University of North Carolina at Chapel Hill, Chapel Hill, NC 27599, USA.

* Both authors contributed equally to this work

**Corresponding author

Victoria L. Bae-Jump MD PhD
Professor
Division of Gynecologic Oncology
University of North Carolina at Chapel Hill
CB# 7572, Physicians Office Building Rm# B105
170 Manning Drive
Chapel Hill, NC 27599. USA
Tel: 919-843-4899
Fax: 919-966-2646,
Email: victoria_baejump@med.unc.edu

Chunxiao Zhou MD PhD
Associate Professor
Division of Gynecologic Oncology
University of North Carolina at Chapel Hill
CB#7295, Lineberger Cancer Center, RM#31-340
450 West Drive
Chapel Hill, NC 27599. USA
Tel: 919-966-3270
Fax: 919-966-2646
Email: czhou@med.unc.edu

Supplementary Table 1 Statistical comparison of IC50 of six cell lines

**Names**	**IC50 (mM) ± SD**	**KLE**	**Hec-1B**	**Ishikawa**	**RL-952**	**AN3CA**	**EC-023**
KLE	1.78 ± 0.26	-	ns	ns	**	*	*
Hec-1B	1.94 ± 0.21		-	ns	**	*	*
Ishikawa	2.95 ± 0.88			-	ns	**	**
RL-952	3.66 ± 0.57				-	**	**
AN3CA	0.48 ± 0.07					-	ns
EC-023	0.33 ± 0.25						-

* p<0.05, ** p<0.01, ns: not significant.

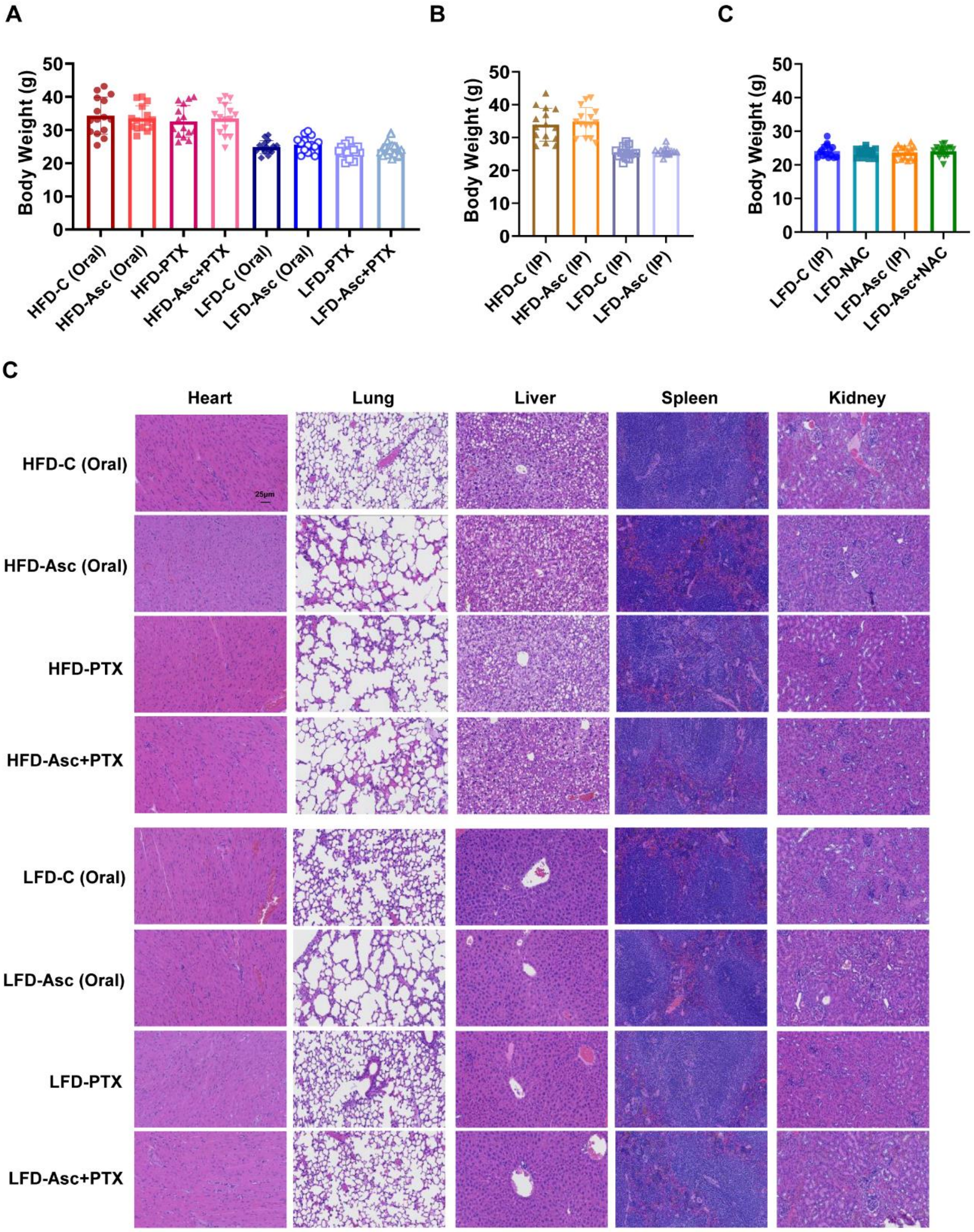

Supplementary Figure 1. The safety profiles of ascorbate in *LKB1$^{fl/fl}$p53$^{fl/fl}$*-transgenic mouse model

The body weight of mice was measured once a week. Results indicate that ascorbate treatment (oral and IP) or combined with paclitaxel for 4 weeks had no significant impact on body weight in both HFD and LFD groups (A, B). The administration of NAC alone or combined with ascorbate (IP) for 4 weeks did not affect the body weight in the LFD group (C). H&E staining

of tissues from the heart, lung, liver, spleen, and kidney demonstrated no morphological changes in major organs after treatment of ascorbate, paclitaxel, and the combination for 4 weeks in the HFD and LFD groups (D). Asc: Ascorbate, HFD: High-fat diet, LFD: Low-fat diet. C (Oral) = Vehicle control for oral administration group, C (IP) = Vehicle control for IP injection group.

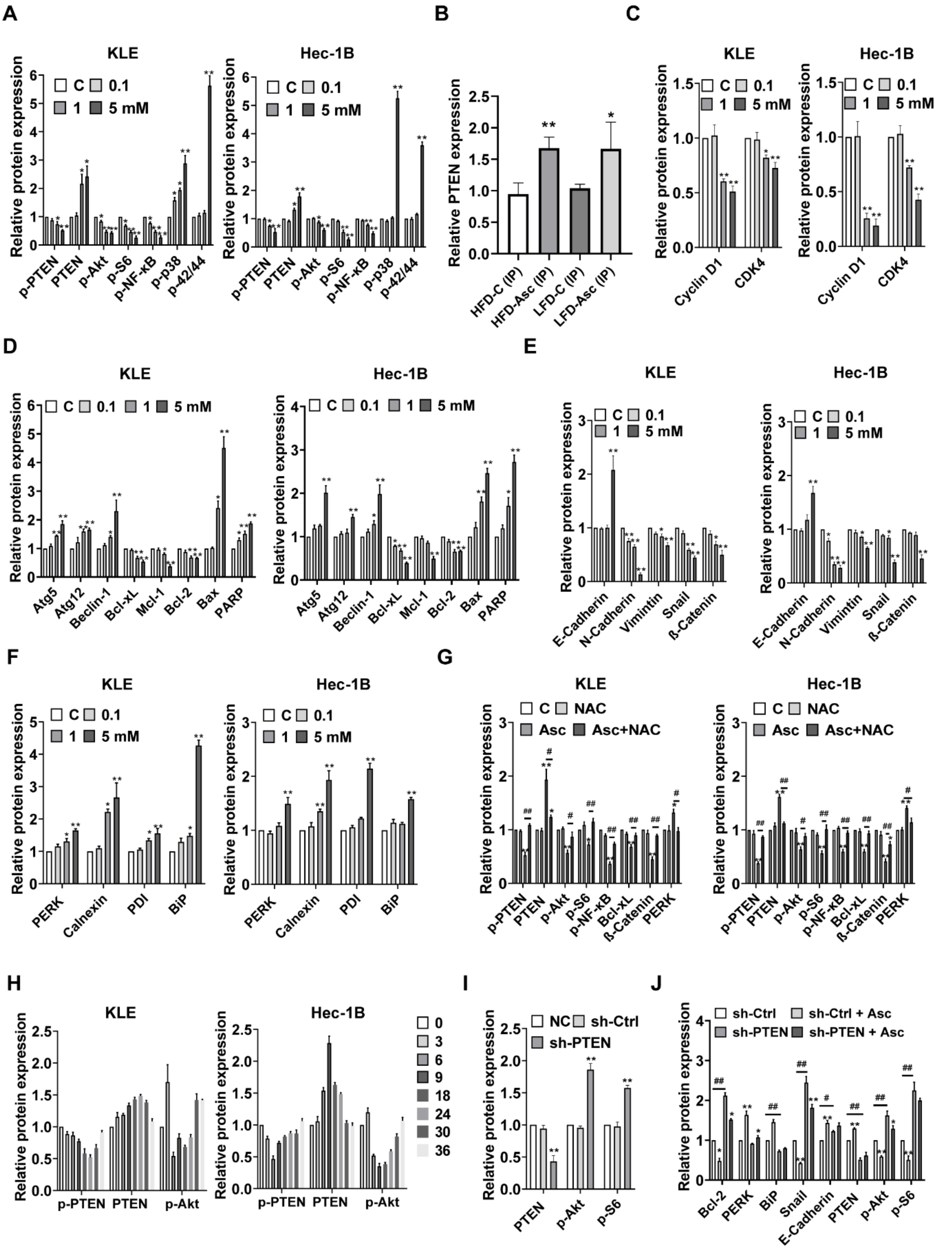

Supplementary Figure 2. The quantitative analysis for Western blotting (Figure 1 to Figure 5)

The quantitative analysis of protein expression for western blotting was conducted using image Lab software and normalized to appropriate internal controls. The quantitative analysis for

Figure 1E (A, B). The quantitative analysis for Figure 2B (C). The quantitative analysis for Figure 2C (D). The quantitative analysis for Figure 3D (E). The quantitative analysis for Figure 4E (F). The quantitative analysis for Figure 4L (G). The quantitative analysis for Figure 5A (H). The quantitative analysis for Figure 5B (I). The quantitative analysis for Figure 5H (J). Asc: Ascorbate, HFD: High-fat diet, LFD: Low-fat diet. C (IP) = Vehicle control for IP injection group. **p*<0.05, ***p*<0.01 compared with C or vehicle control. $^{\#}$*p*<0.05, $^{\#\#}$*p*<0.01 compared with each group.

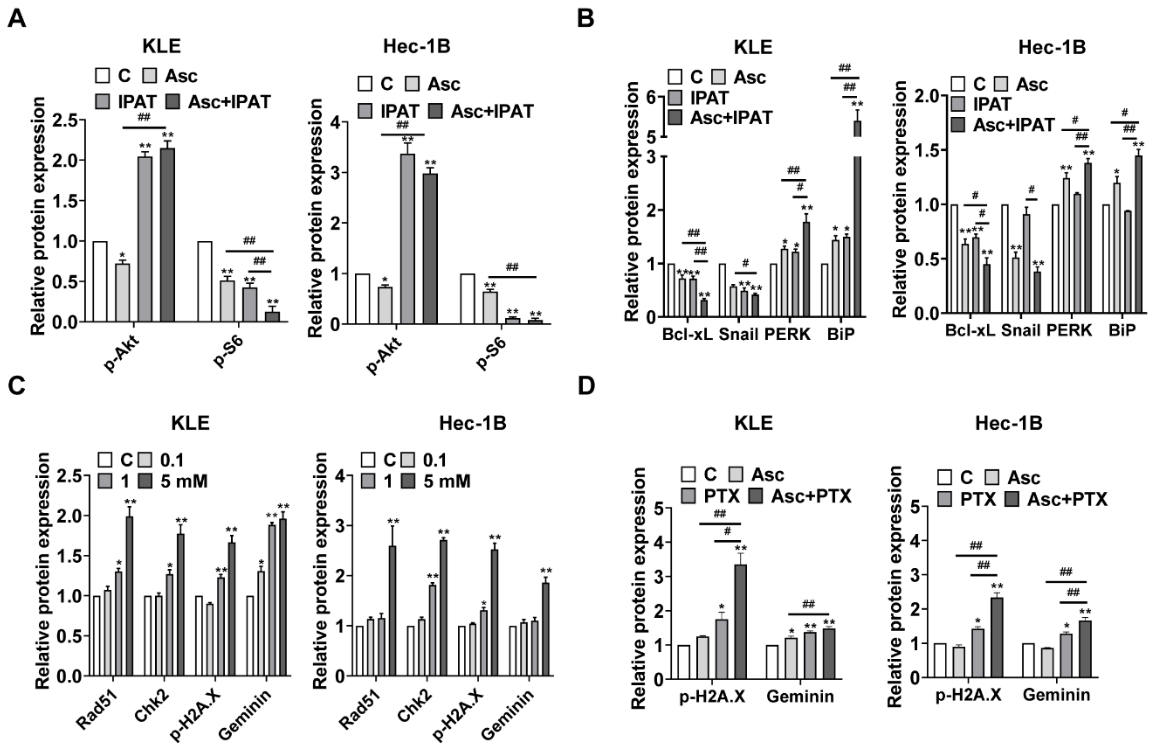

Supplementary Figure 3. The quantitative analysis for Western blotting (Figure 6 to Figure 7)

The quantitative analysis of protein expression for western blotting was conducted using image Lab software and normalized to appropriate internal controls. The quantitative analysis for Figure 6D (A). The quantitative analysis for Figure 6H (B). The quantitative analysis for Figure 7A (C). The quantitative analysis for Figure 7F (D). Asc: Ascorbate, **p*<0.05, ***p*<0.01 compared with C. $^{\#}$*p*<0.05, $^{\#\#}$*p*<0.01 compared with each group.
